# Decoding Solvent Effects in Electrocatalytic Biomass Valorization: Levulinic Acid to γ‑Valerolactone

**DOI:** 10.1021/acssuschemeng.5c12833

**Published:** 2026-04-08

**Authors:** Pol Vilariño, Queralt Bautista, Elvira Gómez, Albert Serrà

**Affiliations:** 1 Grup d’Electrodeposició de Capes Primes i Nanoestructures (GE-CPN), Departament de Ciència de Materials i Química Física, 16724 Universitat de Barcelona, Martí i Franquès, 1, E-08028 Barcelona, Catalonia, Spain; 2 Institute of Nanoscience and Nanotechnology (IN2UB), Universitat de Barcelona, 08028 Barcelona, Catalonia, Spain

**Keywords:** Biomass valorization, Electrocatalytic hydrogenation, Solvent effects, Copper−nickel catalysts, Green chemistry

## Abstract

Electrocatalytic hydrogenation (ECH) of biomass-derived levulinic acid (LA) offers a sustainable route to prepare γ-valerolactone (GVL), a versatile green solvent and fuel additive. Yet despite its promise, most studies overlook the decisive role of the solvent environment, conflating conversion with true product yield. Here we disentangle these effects by systematically probing LA reduction over GC, Cu, Ni, and CuNi cathodes in three contrasting solvents (MeOH, DMSO, IPA) at two temperatures (15 and 35 °C). A clear design rule emerges: the solvents dictate the conversion ceiling, while temperature gates selectivity. At 15 °C, LA consumption is observed but productive lactonization toward GVL remains “off”, yielding only traces of HVA. At 35 °C, lactonization is unlocked, enabling GVL selectivity’s >90% in MeOH with metal-earth-abundant, Ni-based catalysts. Solvent characterization (viscosity, dielectric constant, ionic conductivity) combined with DFT analysis provides a direct rationale for the experimental trends. Methanol emerges as the most effective medium, consistent with its low viscosity and high electrolyte conductivity, which together mitigate diffusion and *iR* penalties relative to IPA and DMSO. DMSO shows intermediate performance, consistent with strong solvation/dielectric stabilization of intermediates, whereas IPA combines high viscosity and low ionic mobility, leading to the lowest conversions. Overall, efficient LA-to-GVL ECH is not dictated by conversion alone but by the coupled interplay of solvent properties, catalyst identity, and temperature required to link surface hydrogenation with thermally assisted lactonization.

## Graphical abstract

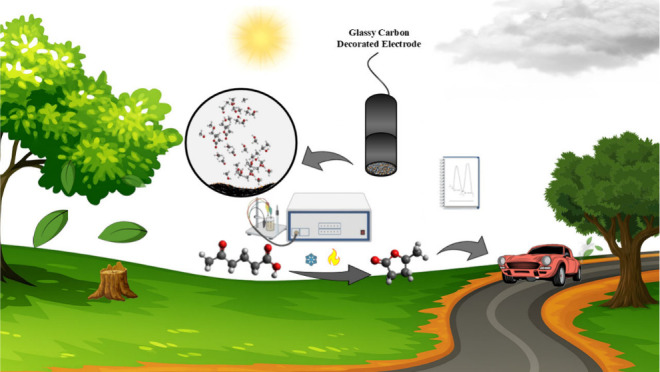

## Introduction

The rising global demand for sustainable energy and chemicals, coupled with the pressing need to address climate change, has intensified the pursuit of renewable and carbon-neutral feedstocks.^1^ Conventional fossil-based processes not only account for substantial greenhouse gas emissions but are also constrained by economic and geopolitical challenges. In this context, biomass emerges as a highly attractive option, offering an abundant and renewable carbon source to produce fuels and chemicals within the frameworks of green chemistry and the circular economy.^1–4^

Among the various molecules derived from lignocellulosic biomass, levulinic acid (LA) stands out due to its high functionality and versatility. Produced via the acid-catalyzed hydrolysis of cellulose and hemicellulose, LA contains both ketone and carboxylic acid groups, making it an ideal precursor for a wide range of high-value chemicals, fuels, plasticizers, and pharmaceutical intermediates. One of the most prominent derivatives is gamma-valerolactone (GVL), which combines low toxicity, biodegradability with diverse applications as a green solvent, fuel additive, and polymer precursor.^5^ Other LA hydrogenation products, such as valeric acid (VA) and 4-hydroxyvaleric acid (HVA), are also gaining attention for these use and application in biodegradable analog of plastics and bioactive compounds, further underscoring LA’s importance in the emerging biobased economy.^4,6–8^

Conventional processes for the hydrogenation of levulinic acid (LA) generally demand harsh operating conditions, such as elevated temperatures and pressures associated with the direct use of hydrogen gas.^9–11^ Moreover, they commonly depend on expensive metal catalysts, including Ru, Pd, or Pt, typically in the presence of externally supplied molecular hydrogen.^8,12–15^ Beyond cost and energy intensity, these conditions introduce nontrivial operability and safety constraints because LA hydrogenation is strongly exothermic and heat-removal limitations can become rate- and safety-limiting at scale. Recent process-safety studies highlight the importance of thermal-mode-aware kinetic modeling and calorimetric assessment for GVL manufacture: for example, adiabatic/isoperibolic analyses emphasize how heat accumulation can drive pronounced temperature excursions, whereas thermal risk assessments quantify thermal-runaway hazards under cooling-failure scenarios and contribute to define safe operating envelopes.^16,17^ These limits sustainability, thereby highlighting the need for alternative catalytic strategies.^18,19^

Electrocatalytic hydrogenation (ECH) emerges as a green and scalable alternative to traditional hydrogenation, operating under milder conditions and utilizing water as a hydrogen source through electrochemical proton reduction.^20,21^ With this approach, ECH, allows for the generation of GVL in situ and provides precise control over reaction kinetics and selectivity through potential modulation, electrode nature and proton concentration. In addition, ECH avoids the need for external hydrogen supply and offers compatibility with renewable electricity sources. Furthermore, recent strategies propose coupling these cathodic transformations with value-added anodic reactions to replace the energy-demanding oxygen evolution reaction, thereby maximizing the overall system efficiency. The development of efficient and robust heterogeneous catalysts remains one of the central challenges in advancing ECH as a viable alternative to conventional hydrogenation methods.^22,23^ Current research efforts in ECH are increasingly focused on identifying catalyst systems that can operate under mild conditions, avoid the use of noble metals, and offer tunable selectivity toward target products such as GVL or HVA. Early electrocatalytic studies have often relied on materials such as lead (Pb) and cadmium (Cd) due to their high overpotentials for hydrogen evolution and apparent selectivity in biomass-derived molecule reduction; however, their toxicity and environmental concerns severely limit their practical applicability.^24,25^ In contrast, transition metal-based systems, particularly those incorporating nickel and copper, have shown promise due to their favorable hydrogenation activity, low cost, and environmental compatibility.^19,26–29^ Despite these advances, the fundamental understanding of how catalyst composition and structure affect activity and product distribution (especially under electrochemical conditions) remains incomplete.^30–32^ Our group has previously demonstrated that CuNi electrodeposited catalysts exhibit good activity and selectivity for LA electroreduction, particularly under acidic aqueous conditions.^33^ The temperatures of 15 and 35 °C were chosen based on prior studies from our group showing that HVA-to-GVL lactonization becomes active above 25 °C, thus allowing us to separately probe hydrogenation (15 °C) and full conversion to GVL (35 °C).^33^ While catalyst design has been investigated, the role of the solvent environment in ECH has received far less attention, protic and aprotic ones. For this our propose is also exploring the influence of organic solvents that could modulate reaction pathways or act as coreactants.^34^ Nonaqueous solvents offer a way to tailor the reaction environment in ECH. Alcohols such as methanol (MeOH) and isopropanol (IPA) can act as hydrogen donors and conductive media, while dimethyl sulfoxide (DMSO), a polar aprotic solvent, provides strong solvation but limited proton availability. Their contrasting properties make them ideal for analyzing how solvent characteristics could influence reactivity and selectivity. These solvents were selected for their distinct physicochemical properties and relevance in green chemistry: IPA and MeOH are renewable alcohols derived from biomass or syngas, while DMSO offers a polar aprotic alternative widely used in catalytic and electrochemical systems.^35–38^

Solvent properties—such as polarity, viscosity, dielectric constant, and hydrogen-bonding ability—can strongly influence reactant solvation, intermediate stabilization, mass transport, and even the competition between hydrogenation and the hydrogen evolution reaction (HER). Polar protic solvents such as IPA and MeOH can promote proton/hydrogen transfer through strong hydrogen-bonding networks and, under acidic conditions, may act as auxiliary H-donors, whereas polar aprotic solvents such as DMSO provide a markedly different solvation environment with distinct ion pairing and interfacial structure. Accordingly, a systematic comparison of these media under identical electrolysis conditions is therefore necessary to elucidate how the solvent governs catalytic performance and reaction pathways. More broadly, solvent choice in (electro)­catalysis does not merely provide a transport medium but directly modulates kinetics and selectivity by controlling dielectric constant, viscosity, and conductivity, which together determine ion mobility, *iR* losses/charge-transfer resistance, and the severity of mass-transport limitations.^39,40^ In addition, solvent–solute and solvent–electrode interactions can reshape adsorption equilibria, hydrogen-bonding networks, and the stabilization of charged or radical intermediates. Because MeOH, IPA, and DMSO can also interact directly with cathode surfaces, solvent identity is expected to affect not only bulk transport but also the interfacial environment (double-layer structure, interfacial proton activity/solvation, and possible competitive solvent adsorption or “site blocking”). In this work, we therefore treat the solvent as an active design variable that tunes the reaction environment; however, we do not claim direct identification of adsorption geometries, which would require operando vibrational spectroscopy and an explicit electrochemical interface framework. Finally, nonpolar solvents (though rarely used in electrocatalysis) generally suppress ionic conductivity and hinder charge transfer, underscoring the need for media that balance polarity, viscosity, and proton availability.^34,41^

Although solvent effects have been considered in selected LA hydrogenation studies, they are often treated implicitly (single-solvent operation) or conflated with catalyst screening, and the solvent is rarely quantified as a design variable through measured transport/electrolyte properties and decoupled temperature effects. Here we adopt a “decoding” strategy: MeOH, IPA, and DMSO are selected not as a broad solvent screen but as a physicochemical contrast matrix (protic vs aprotic; low vs high viscosity; different conductivity and effective proton availability) to disentangle whether conversion ceilings and selectivity are governed primarily by proton delivery, *iR*/transport limitations, or solvation/intermediate stabilization. This approach enables transferable medium-engineering design rules rather than solvent-specific observations. Accordingly, we investigate the electrocatalytic hydrogenation of LA on GC, Cu, Ni, and CuNi cathodes across these three organic solvents spanning contrasting media properties. To rationalize solvent-dependent activity and selectivity, we additionally quantify key solution parameters (most notably viscosity and ionic conductivity) thereby linking performance trends to measurable transport and electrolyte characteristics.

## Experimental Section

### Electrosynthesis of Catalysts

Three electrochemical baths (Table S1) were freshly prepared for the electrodeposition of Cu, Ni and CuNi catalysts following the protocol that has been previously established in our group. The reactants were NiCl_2_·6H_2_O (0.30 M, Merck, 99.9%, CAS: 7791-20-0), CuCl_2_·2H_2_O (0.05 M, >99.0%, CAS: 10125-13-0) as metal source, trisodium citrate dihydrate (Na_3_C_6_H_5_O_7_·2H_2_O) (0.20 M, Sigma-Aldrich, >99.0%, CAS: 6132-04-3) as complexing agent, and NaCl (0.05 M, Sigma-Aldrich, >99.0%, CAS: 7647-14-5) as supporting electrolyte. All solutions were prepared with deionized Milli-Q water, adjusted to pH 3.0 with HCl, and maintained at 25 °C during deposition. Catalysts (Cu, Ni, CuNi) were prepared by electrodeposition by electrodeposition using glassy carbon as the substrate.

Electrodeposition of Cu, Ni, and CuNi catalysts was carried out in a three-electrode cell (glassy carbon, Pt counter, Ag|AgCl|Cl^–^ reference) under argon using potentiostatic control. Detailed procedures and characterization of the resulting films have been reported previously.^33^

### Solvent Properties and Characterization

The properties of the selected solution media (Table S2) were determined the viscosity (η) of solvent–levulinic acid-electrolyte (Et_4_NBF_4_ 0.1 M and H_2_SO_4_ 0.5 M) systems were measured using an Ostwald viscometer (Z275298, Sigma-Aldrich). The determinations were performed at controlled temperatures of 15 and 35 °C using a thermostatic bath, and each reported value corresponds to the average of three independent and reproducible measurements. Ionic conductivity (κ) was evaluated with a conductometer (MT30671562, Mettler Toledo) equipped with a temperature-compensated conductivity cell. Calibration was performed with standard KCl solutions before each series of experiments. Conductivity values were recorded at 15 and 35 °C for both pure solvents and solvent–levulinic acid-electrolyte (Et_4_NBF_4_ 0.1 M and H_2_SO_4_ 0.5 M) systems. Measures were replicated at least three times to ensure reproducibility.

### Electrocatalytic Hydrogenation Tests and Product Analysis

The electrocatalytic performance of the prepared Cu, Ni, and CuNi catalysts was systematically evaluated across the different organic media (MeOH, IPA, and DMSO) using a fixed concentration of 0.50 M levulinic acid. All preparative electrolysis experiments were performed with 0.50 M LA in 10 mL of solvent/electrolyte (i.e., 5.0 mmol LA per run), containing 0.10 M Et_4_NBF_4_ and 0.50 M H_2_SO_4_ (Table S2). Levulinic acid (Sigma-Aldrich, ≥98.0%, CAS: 123-76-2) was prepared in MeOH (Merck, ≥99.85%, ≤0.1% water, CAS: 67-56-1), IPA (Merck, ≥99.5%, ≤0.2% water, CAS: 67-63-0), and DMSO (Sigma-Aldrich, ≥99.9%, ≤0.1% water, CAS: 67-68-5). After electrolysis, LA, GVL, VA, and HVA concentrations were quantified by HPLC (Aminex HPX-87H for LA/VA/HVA; XBridge C18 for GVL), and conversion and Faradaic efficiencies were calculated from these absolute concentrations (Tables S5 and S6). To ensure sufficient ionic conductivity, tetraethylammonium tetrafluoroborate (Et_4_NBF_4_, Thermo Scientific Chemicals, ≥99.0%) was used as the supporting electrolyte at a concentration of 0.10 M (see Table S1). All LA solutions were freshly prepared and maintained under inert conditions prior to electrochemical measurements. To assess the ECH behavior of LA on GC and electrodeposited materials, linear sweep voltammograms (LSV) were carried out between 0.00 V and −2.00 V at 50 mV s^–1^, both in presence and absence of LA. The potentials applied in subsequent chronoamperometric experiments of the ECH process were chosen according to the features observed in these voltammetric profiles. The experiments were performed at 15 and 35 °C under continuous argon bubbling to maintain an inert atmosphere and stir the solution. Because Ar bubbling provides only mild, nonparameterizable convection, we do not attempt an absolute mass-transport model and restrict discussion to comparative scaling based on η and κ.

Surface chemical states were analyzed by X-ray photoelectron spectroscopy (XPS; PHI Quantera SXM, Al Kα) on CuNi electrodes before and after catalytic tests to assess surface integrity and possible changes in the Ni and Cu chemical environments, while elemental composition was examined by field-emission SEM (JEOL JSM-7100F) equipped with energy-dispersive X-ray spectroscopy (EDS/EDX).

After electrolysis, the solution was collected, filtered, and subjected to composition analysis. The liquid phase was further examined by high-performance liquid chromatography (HPLC) with UV–vis detection at 210 nm. GVL was quantified on a reverse-phase XBridge C18 column (3.5 μm, 4.5 × 50 mm) under isocratic conditions with a water:acetonitrile (9:1, v/v) mobile phase and a 10 μL injection loop. The analysis of LA, VA, and HVA was carried out using an Aminex HPX-87H column (300 × 7.8 mm) maintained at 60 °C, employing 10 mM H_2_SO_4_ in water:acetonitrile (9:1, v/v) as eluent, with a 50 μL injection volume.

Conversion was calculated from the HPLC-determined LA concentration as

$$\mathrm{Conversion}\left(\%\right)=\frac{{\left[\mathrm{LA}\right]}_{0}-{\left[\mathrm{LA}\right]}_{t}}{{\left[\mathrm{LA}\right]}_{0}}\times 100$$

FE was calculated from the moles of GVL, VA, and HVA quantified by HPLC (see Supporting Information) and thus represents liquid-phase faradaic closure only, excluding gaseous H_2_ and other unquantified products.

Lactonization was experimentally probed using 4-HVA (Sigma-Aldrich, >99%) as a model intermediate. 4-HVA (0.05 M) was introduced into the standard electrolyte (0.10 M Et_4_NBF_4_ and 0.50 M H_2_SO_4_ in MeOH or IPA) and incubated under stirring at controlled temperature (15 or 35 °C). Aliquots were withdrawn after 1 and 8 h, quenched, and prepared for NMR analysis. ^1^H NMR (400 MHz, CDCl_3_) was used to monitor the conversion qualitatively and by integration of nonexchangeable resonances, following the diagnostic methine signals of 4-HVA (δ 3.85 ppm) and GVL (δ 4.52 ppm). ^1^H NMR spectra were recorded on a Bruker Avance 400 MHz spectrometer at 298 K. Chemical shifts (δ) are reported in ppm relative to TMS or the residual solvent signal. The key resonances used for identification were: 4-HVA: δ 3.85 (m, 1H, CH–OH), 2.45 (t, 2H, CH_2_–COOH), 1.80 (m, 2H, CH_2_), 1.15 (d, 3H, CH_3_); GVL: δ 4.52 (m, 1H, CH–O–C­(O)), 2.55 (m, 2H, CH_2_–C­(O)), 1.90–2.35 (m, 2H, CH_2_), 1.41 (d, 3H, CH_3_).

### Computational Methods

Quantum chemical calculations were carried out with Spartan (Wave function Inc.) to compare with the experimental observations and provide molecular-level insight into the electrocatalytic hydrogenation of LA. All geometries of reactants, and products (LA, GVL, VA, and HVA) were optimized in the gas phase at the M06-2X/6-31+G­(d,p) level of theory. Frequency calculations were performed at the same level to confirm the nature of the stationary points: minima (no imaginary frequencies) or transition states (a single imaginary frequency). Zero-point energy (ZPE) and thermal corrections were included to obtain enthalpies (H°) and Gibbs free energies (G°) at 298 K.^42–44^

## Results and Discussion

### Nonaqueous Solvents as Active Design Variable in Electrocatalytic Hydrogenation of LA

At first sight, investigating nonaqueous solvents for the electrocatalytic hydrogenation of LA to GVL may appear unnecessary. Aqueous electrolytes are inexpensive, safe, and well established, whereas organic solvents require supporting salts, add cost, and raise sustainability questions about recycling and toxicity. If temperature alone controlled GVL selectivity, nonaqueous operation could be dismissed as an academic exercise. Yet, this perspective underestimates the profound influence of solvent on both product distribution and Faradaic efficiency (FE). In water, the HER dominates electron consumption, drastically lowering the FE toward LA reduction. By contrast, solvents such as MeOH, IPA, and DMSO suppress HER, tune proton availability, and stabilize intermediates, thereby channeling a larger fraction of the applied charge into productive hydrogenation. Alcohols can additionally serve as hydrogen donors, while polar aprotic media widen the electrochemical window and mitigate catalyst degradation. Thus, solvent is not a passive carrier but a critical design variable that governs conversion ceilings, selectivity, and FE. Establishing these solvent–temperature design rules provide a foundation for developing efficient, non-noble metal electrocatalysts for biomass valorization.

The selection of MeOH, IPA, and DMSO was therefore guided by their contrasting physicochemical features and their relevance to biomass valorization. Methanol is used here as a benchmark protic solvent because its low viscosity and relatively high conductivity provide a permissive medium for controlled mechanistic comparison across electrodes and temperatures. We acknowledge that MeOH is toxic; however, its hazards are well understood and manageable under closed handling, and it can be efficiently recovered and recycled in process settings. In addition, methanol can in principle be sourced from renewable carbon (e.g., via biomass-derived syngas or CO_2_ hydrogenation), which makes it a relevant candidate for circular process concepts. Importantly, the design rules developed here are not specific to MeOH and provide a framework that can be extended to less hazardous protic media (e.g., ethanol) and other bioderived solvent/cosolvent systems in future work. Isopropanol, more viscous but widely employed in catalytic transfer hydrogenation, is also available via biobased fermentation routes, providing a renewable alcohol framework in addition to its role as a hydrogen donor. DMSO, in turn, provides a polar aprotic medium with a high dielectric constant and strong solvation of reactive species, yet negligible intrinsic proton availability, serving as a mechanistic counterpoint to the alcohols. Having defined this rationale, it becomes essential to examine the physicochemical properties of these solvents. Characteristics such as viscosity, dielectric constant, and ionic conductivity directly influence diffusion, solvation, and charge transport, thereby shaping the conditions under which electrocatalytic reactions proceed. A systematic comparison of these parameters establishes the framework for interpreting how different media affect the transformation of levulinic acid to γ-valerolactone.

#### Viscosity-Driven Mass Transport Limitations

The importance of solvent viscosity in electrocatalysis is often invoked, yet its true impact on performance can be debated. Industrial electrosynthesis is rarely limited by small differences in liquid viscosity once agitation, flow, or porous electrodes are introduced, and critics may argue that measuring viscosities of laboratory solutions adds little beyond confirming textbook expectations. Nevertheless, measuring viscosity does offer a consistent baseline to compare solvents under identical conditions. In this study, the three solvents with contrasting viscosities were examined at 15 and 35 °C (Table 1). Viscosity values were determined for the pure solvents, for solutions containing the supporting electrolyte (Et_4_NBF_4_, 0.1 M and H_2_SO_4_ 0.5 M), and for LA–electrolyte mixtures.

**Table 1:** Viscosity Properties and Ionic Conductivity of Pure Solvents and LA-Containing Solutions^a^

	Viscosity at/mPa s		Conductivity/μS cm^–1^	
Medium	15 °C	35 °C	15 °C	35 °C
DMSO	1.955	1.182	3.35	3.67
DMSO–Electrolyte	2.751	1.655	1965	2038
DMSO–LA–Electrolyte	3.132	1.898	1962	2076
IPA	2.019	1.487	52.4	40.4
IPA–Electrolyte	2.666	2.001	2617	2566
IP −LA–Electrolyte	3.237	2.226	2780	2628
MeOH	0.711	0.325	19.9	8.4
MeOH–Electrolyte	0.736	0.399	37080	26526
MeOH–LA–Electrolyte	0.741	0.410	38000	26800

a LA 0.5 M, Et_4_NBF_4_ 0.1 M and H_2_SO_4_ 0.5 M.

From a critical perspective, one could argue that differences of only a few milli-Pascal seconds in solvent viscosity may seem too small to decisively influence complex electrocatalytic reduction pathways. Nevertheless, viscosity provides a fundamental descriptor of molecular diffusion and the formation of concentration gradients at the electrode interface. Elevated viscosity is expected to hinder mass transport and promote local accumulation or depletion of species, whereas lower viscosity should facilitate more efficient substrate delivery and product removal. Whether such modest physical variations translate into measurable consequences for electrocatalytic performance is precisely the question addressed in this study.

To provide a quantitative (semiempirical) link between viscosity and mass transport, we apply a Stokes–Einstein-type scaling, in which diffusivity is inversely proportional to viscosity (D ∝ 1/η) at a fixed effective hydrodynamic size. We note that solvent-specific solvation and ion pairing can modify the hydrodynamic radius of LA and the dominant charge carriers, so the resulting values should be regarded as first-order relative estimates rather than absolute diffusivities. Using the measured viscosities of the LA–electrolyte mixtures in Table 1, the estimated relative diffusivities are IPA/MeOH ≈ 0.23 at 15 °C and 0.18 at 35 °C, and DMSO/MeOH ≈ 0.24 at 15 °C and 0.22 at 35 °C—corresponding to a approximately 4–5× lower diffusivity in IPA/DMSO relative to MeOH. Because our experiments employ Ar bubbling (rather than a hydrodynamically defined boundary layer), we treat this as a comparative scaling rather than a predictive mass-transport model; nevertheless, it is consistent with the markedly smaller LA–blank current increments in IPA/DMSO (Table S3) and the longer times required to pass the same total charge under otherwise identical conditions (Table S4).

It is noteworthy that the addition of the mixed electrolyte (0.1 M Et_4_NBF_4_ + 0.5 M H_2_SO_4_) increases the viscosity of DMSO substantially more than that of MeOH (Table 1). For example, at 15 °C the viscosity rise is Δη ≈ +0.80 mPa s in DMSO (1.955 → 2.751 mPa s) versus only Δη ≈ +0.03 mPa s in MeOH (0.711 → 0.736 mPa s), and a similar trend holds at 35 °C (DMSO: + 0.47 mPa s; MeOH: + 0.07 mPa s). This behavior is consistent with stronger and longer-lived ion–solvent solvation in highly dipolar DMSO, which increases the effective hydrodynamic size of the charge-carrying species (and may promote ion-paired/associated structures), thereby increasing viscous drag. In contrast, methanol’s low baseline viscosity and faster solvent-exchange dynamics lead to a smaller net increase in bulk viscosity upon electrolyte addition.

#### Solvent Polarity, Solubility, and Electron Transfer Kinetics

While solvent polarity and dielectric constant are frequently cited as key determinants of electron-transfer kinetics, their direct impact on complex electrocatalytic reductions is not always straightforward. From a skeptical perspective, one might argue that differences in dielectric constant between common organic solvents are unlikely to outweigh other factors such as electrode surface chemistry, applied potential, or local proton availability. In addition, the solubility of LA and its intermediates can be at least as influential as dielectric stabilization in determining the effective concentration of reactants at the electrode interface. If solubility varied significantly across solvents, apparent activity differences could simply reflect substrate availability rather than intrinsic effects on charge-transfer kinetics. In practice, however, LA exhibits high solubility in all three solvents studied (∼0.9 kg L^–1^ in MeOH, ∼0.8 kg L^–1^ in propanols, ∼0.1 kg L^–1^ in DMSO), excluding solubility limitations under the present conditions.^45^ Polarity therefore remains a useful descriptor of how the solvent reorganizes around charged or partially charged species during electron transfer. DMSO, with its high dielectric constant and dipole moment, facilitates dipole reorientation and stabilizes charge redistribution, features often invoked to rationalize its electrochemical utility. Protic solvents such as methanol and isopropanol, in contrast, form extended hydrogen-bond networks that rigidify the solvation shell and could increase the reorganization barrier, potentially slowing charge-transfer events. Whether these polarity effects dominate over contributions from solubility or mass transport remains debatable, but systematic comparison of solvents with sharply contrasting properties is essential to disentangle their relative influence.

Although solvent-dependent kinetics are sometimes rationalized using a Marcus-type reorganization-energy framework, we do not attempt to extract quantitative reorganization energies (λ) here, as this would require explicit interfacial/constant-potential modeling and solvent-phase transition-state treatments. Instead, we interpret solvent effects through a coupled transport–solvation picture constrained by experimentally measured viscosity and ionic conductivity (Table 1), supported by first-order comparative scaling (D ∝ 1/η; expected R_u_ ∝ 1/κ for fixed cell geometry), and anchored in steady-state electrolysis outcomes (conversion, FE, and selectivity). In MeOH, the combination of low viscosity and very high conductivity in the acid/salt electrolyte minimizes *iR* and concentration overpotentials and provides a protic hydrogen-bonding environment that supports interfacial proton/hydrogen transfer. In IPA, higher viscosity and lower effective ion mobility amplify transport and interfacial sensitivity, making selectivity more susceptible to potential and double-layer perturbations. In DMSO, strong dielectric solvation can stabilize polar intermediates, yet its aprotic nature shifts proton delivery/solvation to acid-derived species and ion-paired structures, which can impose kinetic constraints and confine selectivity to narrower operating windows.

#### Ionic Conductivity and Ohmic Resistance

Ionic conductivity is frequently invoked as a critical factor in electrocatalysis, yet its interpretation in nonaqueous systems is not without ambiguity.^9,46^ From a critical perspective, it could be argued that that conductivity values in organic solvents largely reflect the presence of the supporting electrolyte rather than intrinsic solvent properties. Indeed, the pure solvents investigated here show conductivities below 100 μS cm^–1^, essentially nonconductive without salt. The dramatic increase observed upon addition of Et_4_NBF_4_—up to ∼3.8 × 10^4^ μS cm^–1^ in methanol-based mixtures—demonstrates that ion mobility is dominated by salt dissociation, and thus any ranking of conductivity may be as much about electrolyte–solvent interactions as about the solvent itself (Table 1).

Nevertheless, the differences between the three solvents remain substantial. Methanol–LA–electrolyte solutions show conductivities 1 order of magnitude higher than those of IPA or DMSO mixtures, suggesting more efficient ion dissociation and transport. The conductivity trends observed upon addition of the mixed electrolyte (0.1 M Et_4_NBF_4_ + 0.5 M H_2_SO_4_) reveal a hierarchy that is not easily reconciled with the neat-solvent background values, which are governed primarily by trace impurities. Although IPA initially displays higher intrinsic conductivity than MeOH, the introduction of the electrolyte reverses this ranking, with MeOH exhibiting conductivities more than an order of magnitude greater than IPA and DMSO. This inversion underscores that neat values are poor predictors of electrolyte behavior, which is instead dictated by the balance between ion dissociation, solvation, and mobility. In MeOH, the combination of moderate permittivity and very low viscosity ensures both efficient ion separation and fast transport of Et_4_N^+^, BF_4_^–^, and the highly mobile proton/hydrogen sulfate species supplied by H_2_SO_4_. By contrast, IPA is penalized by its low dielectric constant, which promotes ion pairing and restricts the number of effective carriers, while DMSO, despite its high permittivity, imposes severe mobility penalties from viscosity and asymmetric solvation, particularly the weak stabilization of BF_4_^–^ and HSO_4_^–^. The slight increase in conductivity upon addition of levulinic acid—despite an increase in viscosity—suggests that enhanced hydrogen-bond donor character may facilitate partial disruption of ion pairing, although the role of water traces introduced with H_2_SO_4_ or LA cannot be discounted. It should also be considered that mixing concentrated H_2_SO_4_ with these solvents can generate new species—such as methyl or isopropyl hydrogen sulfates, ethers, or sulfoxonium salts—whose ionic character may further contribute to the measured conductivity. In this sense, the exceptionally high conductivities observed in MeOH media may not arise solely from favorable solvent polarity and viscosity, but also from the disproportionate contribution of proton transport, acid–solvent reaction products, and water-mediated microdomains. Whether such disparities in bulk conductivity translate into differences in interfacial charge delivery is less certain, since local electric fields, double-layer structure, and electrode roughness can strongly modulate effective proton concentrations at the reactive interface. Critics might contend that bulk conductivity measurements alone cannot predict actual interfacial conditions, and that ohmic losses in a small laboratory cell are unlikely to be limiting. Even so, conductivity provides a useful comparative descriptor of solution behavior under otherwise identical conditions. Higher conductivity should, in principle, minimize uncompensated resistance (*iR* drop), improve potential control, and sustain more reliable charge delivery to the electrode surface. Conversely, lower conductivity is expected to increase resistive losses and potentially exacerbate parasitic processes. A simple way to translate conductivity into potential-control implications is to note that the solution resistance (and, by extension, the expected *iR* sensitivity) scales inversely with conductivity for a fixed cell geometry. Using the κ values of the LA–electrolyte mixtures (Table 1), IPA and DMSO are expected to exhibit ∼ 14× and ∼ 19× higher solution resistance than MeOH at 15 °C, and ∼10× and ∼13× higher at 35 °C. Thus, for a given current, *iR* losses and potential-control errors are intrinsically more severe in IPA/DMSO, which can suppress observable charge throughput and distort comparisons based on voltammetric current magnitude. We emphasize that absolute R_u_ depends on electrode spacing and cell geometry; the purpose of this scaling is to rationalize relative solvent trends rather than to claim a fully predictive transport model. Whether these effects outweigh mass-transport limitations or solvation dynamics remains an open question, but systematic evaluation of conductivity across solvents offers a necessary reference point for discussing observed electrocatalytic trends.

### Electrocatalytic Hydrogenation of LA in Nonaqueous Solvents

Having and examined the key physicochemical properties of MEOH, IPA, and DMSO, the next step is to assess how these solvents influence the electrocatalytic hydrogenation of LA.

LSV was employed to gauge the electrochemical response of LA in MeOH, IPA, and DMSO on GC, Cu, Ni, and CuNi electrodes, with and without LA (Figure 1). The preparation and characterization of the electrodes have been described in detail in our previous publication. At first glance, the increase of cathodic currents observed upon adding LA—especially in methanol—could be interpreted as evidence of facile LA reduction across media. A more critical view, however, recognizes several factors that limit how directly LSV currents can be mapped onto substrate-specific kinetics.

**Figure 1:**
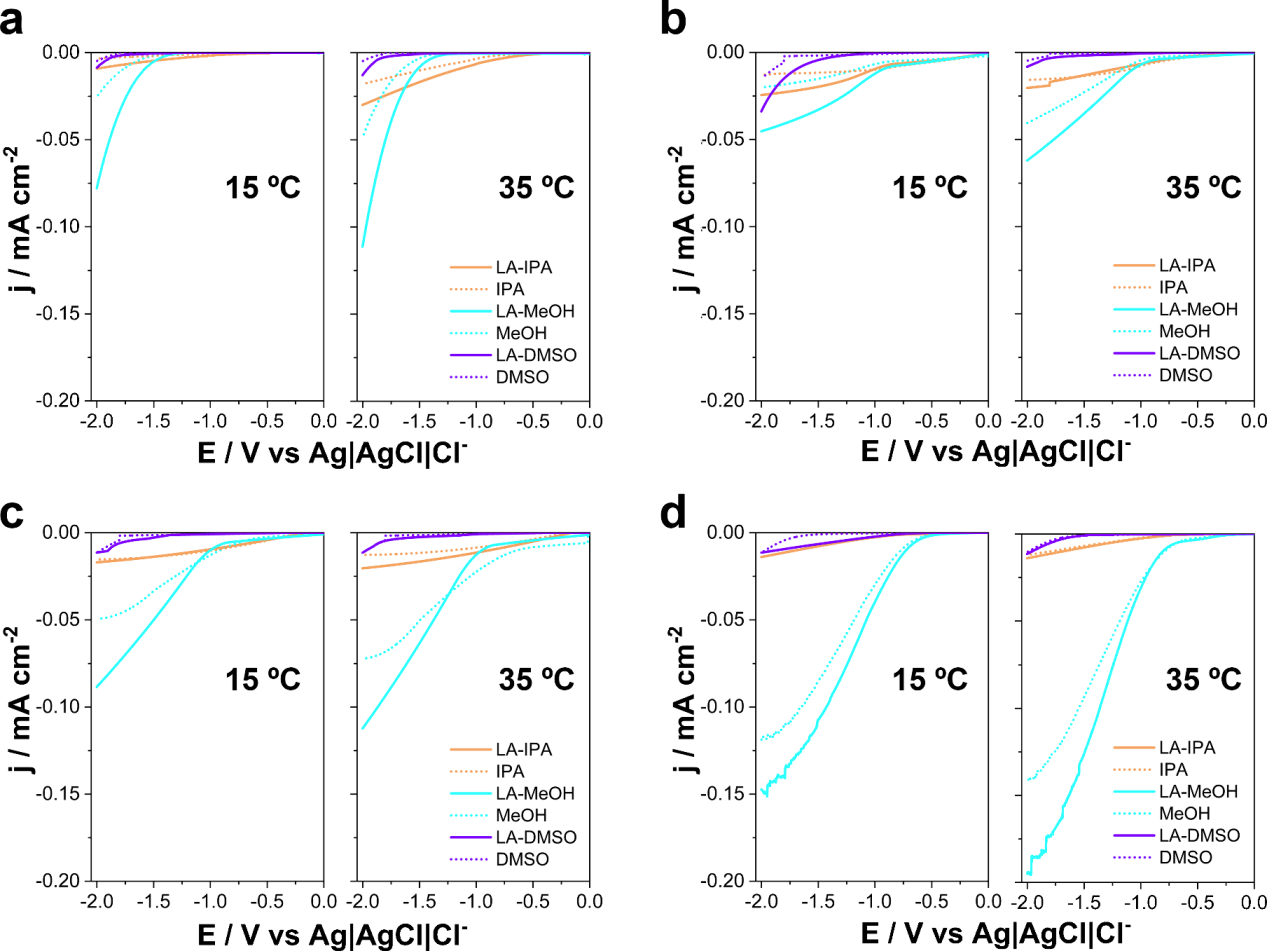
Linear sweep voltammograms of (a) GC, (b) Cu, (c) Ni, and (d) CuNi electrodes recorded in MeOH, IPA, and DMSO electrolytes containing 0.50 M LA, 0.10 M Et_4_NBF_4_, and 0.50 M H_2_SO_4_, along with the corresponding blanks (all components except LA). Measurements were performed at 15 °C (left panels) and 35 °C (right panels) with a scan rate of 50 mV s^–1^.

Remember that bulk conductivity and viscosity differ by more than an order of magnitude between the media after electrolyte addition (Table 1). As a result, uncompensated resistance and diffusion-layer thickness vary substantially from solvent to solvent. Higher |j| in methanol can therefore reflect lower *iR* drop and thinner diffusion layers rather than intrinsically faster LA electron transfer. This observation underscores that macroscopic solvent properties (specifically viscosity and ionic conductivity) can effectively mask intrinsic catalytic trends in LSV, cautioning against the use of simple current magnitude as a direct proxy for turnover frequency. Accordingly, we refrain from extracting kinetic parameters from LSV and use these scans only as a qualitative indicator of substrate-induced current response; solvent comparisons are instead anchored in controlled electrolysis metrics (conversion, FE, and product distribution) obtained under fixed charge and identical cell configuration/hydrodynamics.

Conversely, the depressed currents in IPA and DMSO are consistent with their lower conductivity and higher effective viscosity, but this points to transport penalties more than to mechanistic inactivity. Because the electrolytes contain 0.10 M Et_4_NBF_4_ and 0.5 M H_2_SO_4_ in organic media, the voltammetric background is shaped by strong electrolyte–solvent interactions (ion pairing, specific adsorption) and possible acid–solvent reactions (e.g., alkyl hydrogen sulfates in alcohols; sulfoxonium species in DMSO). These processes alter the double-layer structure and may introduce parallel Faradaic pathways. Apparent onset shifts or current increases upon adding LA can therefore arise from changes in interfacial composition and proton availability rather than solely from LA reduction (especially when these changes are small).

The onset potentials extracted from the LSVs deserve careful scrutiny. At first glance, the earlier onset observed in MeOH, particularly on Ni and CuNi, might be taken as evidence of more favorable electron-transfer kinetics or stronger substrate–catalyst interactions. Yet such an interpretation risks conflating transport accessibility with intrinsic kinetics. Lower viscosity and higher ionic conductivity in MeOH reduce solution resistance and facilitate proton availability, which can shift apparent onsets positively without any fundamental change in the reaction barrier. Conversely, the more negative onsets recorded in IPA and DMSO may reflect ohmic drop, sluggish proton transfer, or diffusion limitations rather than a genuine increase in activation energy to start reaction. Thus, while onset potential shifts are a useful comparative descriptor of media effects, they cannot be uncritically ascribed to catalytic activity.

The voltammetric current difference between LA-containing and blank solutions at fixed potential, Δj = j­(LA) – j­(blank), is often presented as a practical diagnostic but not a definitive kinetic metric (Table S3). At −1.80 V, Δj is most negative in MeOH for all electrodes and temperatures, consistent with its favorable κ/η profile. Using the LA–electrolyte properties in Table 1, a Stokes–Einstein-type scaling (D ∝1/η) suggests ∼4–5× lower relative diffusivity in IPA/DMSO than in MeOH, while the measured conductivity differences imply ∼10–20× higher expected solution resistance (and thus greater *iR* sensitivity) for a fixed cell geometry. Together, these factors can substantially reduce the registrable Δj in IPA/DMSO even if the intrinsic interfacial reactivity were similar. However, this interpretation risks oversimplification. Parasitic pathways—hydrogen evolution, acid–solvent side reactions, or adsorption phenomena—can affect the cathodic response at the chosen overpotential. In other words, “more current” does not necessarily mean “more LA reduction”.

In all the conditions explored, MeOH displays the most consistent enhancement upon LA addition, with |Δj| values typically ranging from 0.018 to 0.029 mA cm^–2^ across electrodes and temperatures. This trend aligns with MeOH’s favorable physicochemical properties which minimize ohmic resistance and diffusion barriers. However, it should be noted that these apparent improvements may not reflect intrinsic catalytic promotion by LA, but rather solvent-mediated differences in charge delivery and mass transport. In sharp contrast, IPA yields very small |Δj| values, often below 0.005 mA cm^–2^, with several cases near the instrumental noise floor. Given IPA’s higher viscosity and lower dielectric constant, these negligible shifts can just as plausibly be attributed to mass-transport and ion-pairing penalties as to any true kinetic suppression of LA reduction. From a critical standpoint, it is difficult to claim mechanistic insight when the LA-induced currents are indistinguishable from background variability. DMSO produces intermediate |Δj| values (0.001 to 0.006 mA cm^–2^).

Electrode identity modulates the absolute current densities, with Ni and especially CuNi generally displaying earlier onsets and higher |j| than GC or Cu. Yet, when Δj is used as the comparison metric, solvent control appears dominant over catalyst effects: in MeOH each electrode shows a measurable LA-dependent increment, while in IPA and DMSO the responses collapse toward zero. Raising T from 15 to 35 °C generally increases |j|, but not uniformly across all solvent–electrode pairs. Given that viscosity drops and conductivity modestly rises with temperature, part of the apparent improvement in current response is attributable to improved transport and reduced uncompensated resistance, rather than to a genuine lowering of intrinsic activation barriers for LA reduction.

Taken together, these results confirm that LA perturbs the cathodic response of all electrodes, but they also highlight the risk of overinterpreting LSV as a proxy for intrinsic reaction kinetics. The solvent-dependent Δj values are more plausibly linked to macroscopic properties—viscosity, conductivity, and proton availability—than to direct evidence of solvent-mediated tuning of electron-transfer barriers. In this sense, LSV serves primarily as a qualitative diagnostic, signaling that MeOH provides the most permissive medium for observing substrate reduction, while IPA and DMSO impose strong transport or solvation penalties. Yet, the true mechanistic picture cannot be extracted from voltammetry alone. A meaningful assessment of solvent effects requires correlating current increments with FE and product distribution under steady-state electrolysis. Only by establishing this connection can one disentangle solvent-driven transport phenomena from genuine catalytic enhancement. With these caveats in mind, the discussion now turns from qualitative LSV signatures to controlled electrolysis, where quantitative selectivity and efficiency metrics provide a solvent-normalized framework for evaluating levulinic acid hydrogenation. Importantly, beyond macroscopic transport and *iR* effects, specific interfacial interactions—including competitive solvent adsorption/coordination and double-layer restructuring—may also contribute to the solvent dependence observed here. Because voltammetry alone cannot disentangle these contributions, we rely on controlled electrolysis (conversion, FE, and product distribution) to assess solvent effects under steady-state conditions.

To ensure meaningful comparisons among catalysts and media, all experiments were conducted under identical total charge (1000 C; Area: 0.126 cm^–2^) throughput. The electrocatalytic performance was systematically evaluated at two applied potentials (−1.60 V and −1.80 V vs Ag/AgCl) and two temperatures (15 and 35 °C) under continuous Ar bubbling, which ensured an oxygen-free environment and provided mild convection to minimize mass-transport limitations. The average electrolysis durations were approximately 10 h in MeOH, 20 h in IPA, and 40 h in DMSO, reflecting the differing kinetic regimes of each medium (Table S4). Because electrolysis in viscous media (especially DMSO) requires substantially longer times, blank-control measurements were used to assess background stability under our conditions. Linear sweep voltammograms of the blank electrolytes (solvent + H_2_SO_4_ + Et_4_NBF_4_, without LA) were recorded at 15 and 35 °C, and no additional faradaic features indicative of bulk solvent/electrolyte decomposition were observed within the working potential window. Moreover, blank solutions held at 35 °C for comparable time scales showed no qualitative change in their voltammetric response, supporting that the dominant solvent effect in viscous media is transport/*iR* and interfacial structuring rather than rapid bulk degradation. Importantly, no systematic current decay indicative of progressive deactivation was observed over these electrolysis durations (including ∼40 h runs in DMSO); after an initial stabilization transient, the cathodic current remained comparatively stable, suggesting the absence of persistent poisoning under the conditions studied. To assess whether the observed solvent dependence could stem from irreversible catalyst degradation or surface poisoning, we performed postelectrolysis EDS and XPS analyses of the CuNi electrodes. The near-surface chemical state of CuNi is preserved after electrolysis in each solvent, with no detectable shifts in the Cu 2p or Ni 2p XPS features (Figure S1). In parallel, EDS confirms retention of a CuNi bimetallic composition. A modest Cu enrichment is observed (ΔCu ≈ +3 at. % under the representative condition), which falls within the expected range for sampling/statistical variability across rough electrodeposits and/or mild surface reorganization/segregation under cathodic polarization; importantly, it does not indicate preferential dissolution of either component that would compromise the bimetallic character. Overall, these results support that the dominant solvent effects reported here are largely reversible and medium-imposed within the explored operating window.

#### Faradaic Efficiency, Conversion, and Product Distribution

The FE (see Supporting Information for full calculation details) values presented here were evaluated only for the three selected hydrogenation products—GVL, VA, and 4-HVA. Conversion refers to the chemical conversion of LA, quantified from LA depletion by HPLC. In contrast, FE quantifies only the fraction of the passed charge recovered in the HPLC-quantified liquid-phase products (GVL, VA, and HVA) and therefore does not imply 100% charge closure. Other charge sinks, including gaseous H_2_ from the hydrogen evolution reaction (HER), volatile/undetected organics, and potential solvent-derived byproducts, are not captured by the LC analysis; consequently, the reported FE values represent a partial charge balance. This distinction is important when interpreting trends: a lower summed FE can reflect greater charge loss to competing pathways (notably HER) and/or formation of unquantified species, whereas a higher summed FE indicates a larger fraction of charge directed toward the quantified products without excluding parallel reactions.

To further constrain interpretation of the “missing charge”, it is useful to consider the charge budget imposed by our fixed-charge protocol. Each electrolysis was carried out at Q = 1000 C in 10 mL electrolyte containing 5.0 mmol of LA, corresponding to 2.07 e^–^ per LA molecule. The initial hydrogenation step (LA → 4-HVA)—and thus the net transformation LA → GVL, given that 4-HVA → GVL is a nonfaradaic lactonization—requires 2 e^–^ per LA. Therefore, if LA depletion proceeded exclusively via this 2e^–^ hydrogenation pathway, complete faradaic conversion of 5.0 mmol LA would require approximately 965 C. Under high-conversion conditions, this provides an upper bound on the charge that could be diverted to HER; for example, at 90% conversion, the maximum residual charge is approximately 130 C, corresponding to ≤0.68 mmol H_2_. This stoichiometric constraint indicates that the unassigned charge fraction (100 minus the summed FE for GVL/VA/4-HVA) cannot be interpreted uniquely as H_2_ formation, and must also include unquantified organic sinks and/or concurrent nonfaradaic LA consumption (e.g., acid-catalyzed esterification in alcohol media, ketalization, or adsorption/retention phenomena). Product distributions (Figure 3 and Tables S5 and S6) are reported as molar selectivities relative to converted LA; accordingly, the “Other products” fraction denotes converted LA not recovered as GVL/VA/4-HVA, and should not be conflated with a charge fraction.

At 15 °C, conversions were generally high (≈50–92%), yet FE values remained below 10% across all solvents and catalysts (Figures 2 and 3 and Tables S5 and S6). This apparent high-conversion/low-FE regime reveals a pronounced decoupling between substrate depletion and electron economy. Because conversion is defined from LA depletion by HPLC, LA “disappearance” does not necessarily imply productive faradaic turnover into the quantified products. Rather, the FE results indicate that most of the passed charge is diverted to parasitic sinks—predominantly HER (H_2_) and potentially including solvent/electrolyte coreduction and volatile/undetected organics outside the LC window—while only a minor fraction is recovered as liquid-phase hydrogenation products (GVL/VA/4-HVA). In parallel, nonfaradaic and/or analytically untracked pathways (e.g., adsorption/retention at interfaces and acid-mediated transformations in organic media) may also contribute to apparent LA depletion and are therefore discussed here as plausible contributors to the conversion–FE mismatch. Under these conditions, product distributions were dominated by 4-HVA (≈9–11%), with essentially no GVL or VA detected, consistent with kinetic suppression of the thermally activated 4-HVA → GVL lactonization at low temperature. Mechanistically, this indicates that low-temperature operation permits LA depletion and intermediate formation but remains energetically inefficient and electrochemically unselective, because charge is largely consumed by competing pathways rather than consolidated into the target product pool. It is crucial to note that this low FE is not due to poor intrinsic catalyst activity; our group has previously demonstrated that these same CuNi catalysts achieve >80% FE in aqueous media at 25 °C.^47^ The drastic drop observed here confirms a solvent-imposed “gating effect”, where the lack of thermal energy and solvent structure physically block the productive lactonization pathway, diverting charge to parasitic sinks like HER.

**Figure 2:**
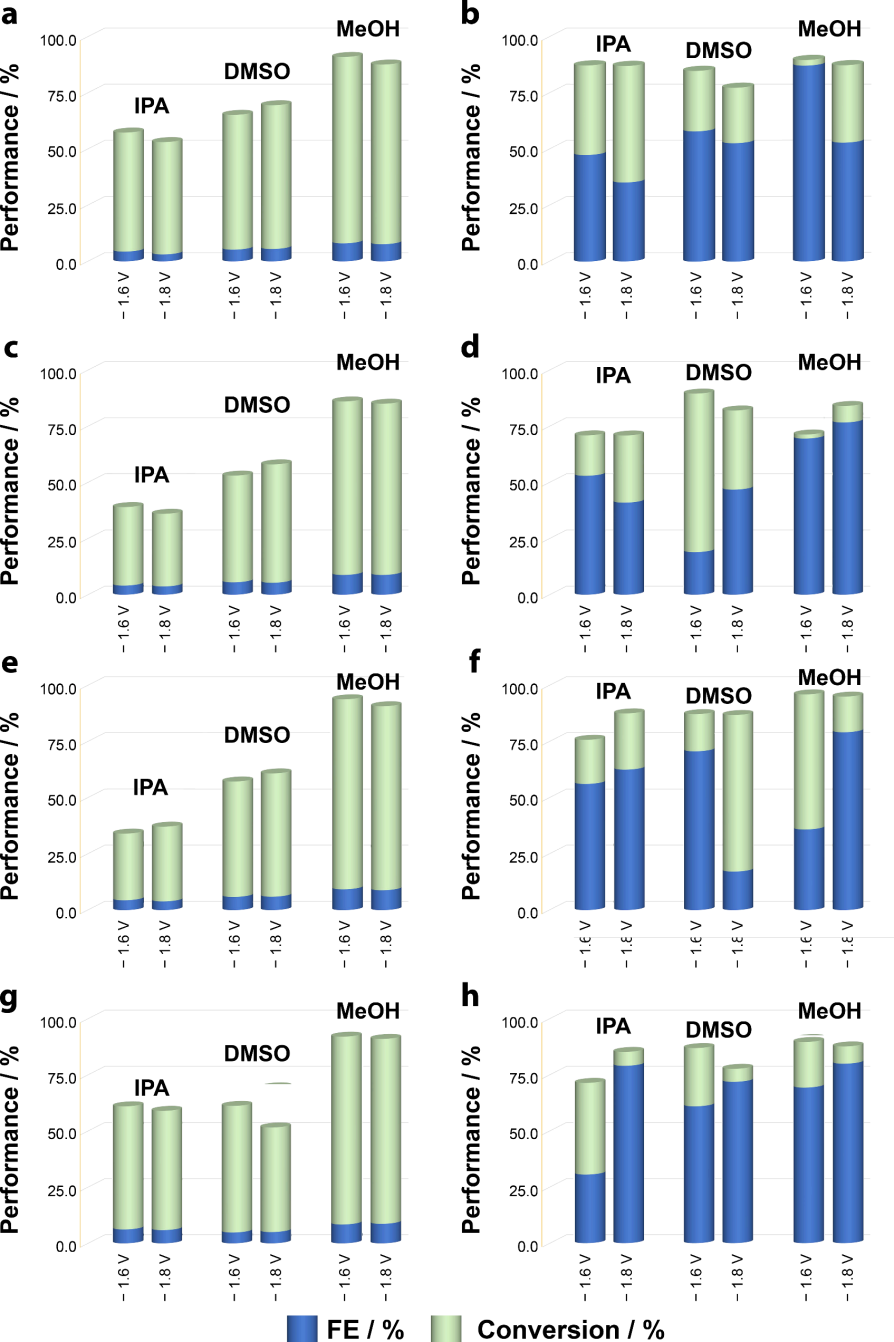
Faradaic efficiency (FE) and levulinic acid (LA) conversion for (a, b) glassy carbon (GC), (c, d) Cu, (e, f) Ni, and (g, h) CuNi electrodes in nonaqueous electrolytes (MeOH, IPA, DMSO) containing 0.50 M LA, 0.10 M Et_4_NBF_4_, and 0.50 M H_2_SO_4_. Panels a, c, e, g correspond to experiments at 15 °C, while panels b, d, f, h correspond to 35 °C. Each electrolysis was conducted under continuous Ar bubbling with a fixed total charge (1000 C; geometric area = 0.126 cm^2^). Conversion refers to LA depletion measured by HPLC, whereas FE accounts only for the summed liquid-phase products (GVL, VA, HVA) and does not include H_2_/volatile or other unquantified sinks.

**Figure 3:**
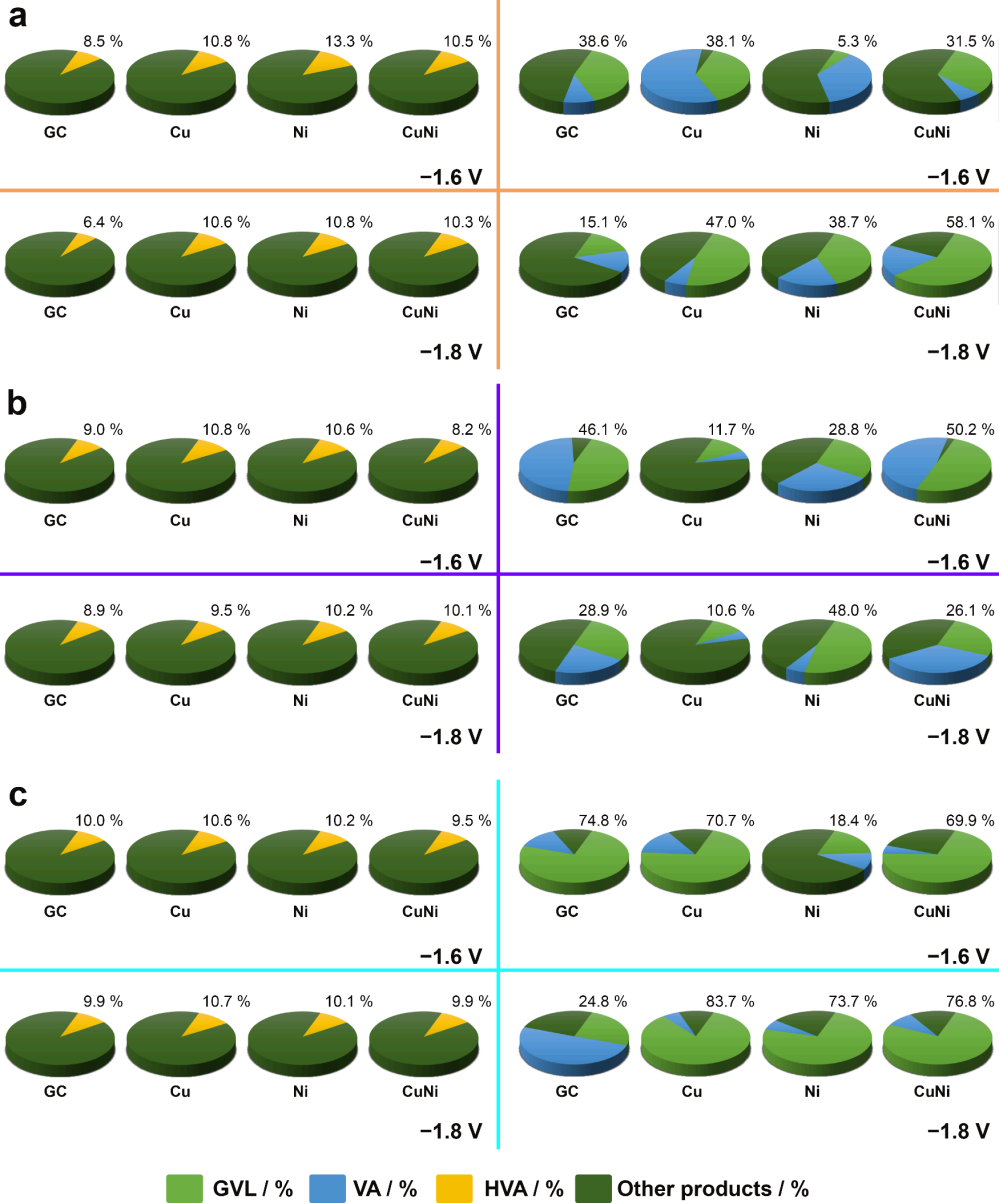
Product distribution of LA electrochemical hydrogenation (ECH) on glassy carbon (GC), Cu, Ni, and CuNi electrodes in nonaqueous electrolytes: (a) IPA, (b) DMSO, and (c) MeOH, each containing 0.50 M LA, 0.10 M Et_4_NBF_4_, and 0.50 M H_2_SO_4_. Left panels show experiments at 15 °C, while right panels show those at 35 °C. Electrolyses were carried out under continuous Ar bubbling with a fixed total charge of 1000 C (geometric area = 0.126 cm^2^). Product distributions are expressed as molar selectivities (% of converted LA) based on HPLC quantification. Accordingly, the “Other products” fraction denotes converted LA not recovered as GVL, VA, or 4-HVA. Faradaic efficiencies for the same product set are reported separately (Figure 2 and Tables S5 and S6); the unassigned charge fraction (100 minus the summed FE for GVL/VA/4-HVA) is attributed to HER and to unquantified liquid/volatile organic sinks (including possible solvent-derived chemistry).

At 35 °C, FE values increased sharply but, in a solvent, and electrode-dependent manner (Figures 2 and 3 and Tables S5 and S6). In methanol, FE up to 80% were achieved (Ni, Cu, CuNi at – 1.8 V), with product distributions dominated by GVL (70–84%). However, the coincidence between improved FE and the onset of thermally assisted lactonization raises the possibility that the “efficiency gain” reflects the consolidation of intermediates into counted products rather than inherently improved electron utilization. Glassy carbon in MeOH at −1.6 V, for instance, delivered 87% FE despite being nominally inert, underscoring that solvent-mediated transport and thermal chemistry may override catalyst-dependent kinetics.

This result underscores that mitigating parasitic charge sinks (e.g., HER, possible solvent/electrolyte coreduction, and competitive adsorption/site blocking) cannot be addressed solely through catalyst selection. Instead, medium engineering—the synergistic optimization of solvent identity and thermal conditions—emerges as a key lever to improve charge partitioning toward the desired hydrogenation products by tuning transport, proton activity, and the interfacial environment. In particular, moving to MeOH at 35 °C biases the kinetic competition toward productive LA hydrogenation and downstream lactonization, while reducing the relative contribution of parasitic pathways; notably, the apparent efficiency gain may also reflect thermal consolidation of intermediates into the quantified product pool (HVA → GVL) under conditions where lactonization becomes accessible. Within the LA → 4-HVA → GVL sequence, GVL selectivity is maximized by operating in a regime where (i) interfacial hydrogenation to 4-HVA proceeds (sufficient proton activity and charge delivery), (ii) lactonization is kinetically accessible (moderate temperature), and (iii) over-reduction is avoided (bounded cathodic bias). In practice, this translates to selecting a solvent/electrolyte combination that minimizes *iR*/transport penalties and supports interfacial proton delivery, while using potentials that favor cyclization over deeper reduction to VA.

In DMSO (at 35 °C), FE values were more variable. At −1.8 V, FE ranged from only 17% on Cu (with >80% of converted LA not recovered as GVL/VA/4-HVA (Table S6)) to 72% on CuNi, where VA dominated (35%) alongside GVL (26%). At −1.6 V, certain cases (e.g., CuNi) approached 61% FE with unusually clean product distributions (GVL ≈ 50%, VA ≈ 48%, unquantified products ≈ 2%). These windows of efficiency suggest that DMSO is capable of supporting selective electroreduction, but only under narrow conditions, while elsewhere its high viscosity and complex solvation behavior amplify charge losses into parallel pathways. Importantly, solvent-switch recovery controls (DMSO exposure → rinsing → IPA/MeOH electrolysis) showed no persistent loss of activity or selectivity relative to pristine electrodes operated directly in MeOH. This observation is consistent with predominantly reversible medium effects (interfacial solvation/double-layer restructuring and transport penalties) rather than irreversible active-site poisoning by DMSO under the conditions used here. In absence of sophisticated and no accessible operando vibrational spectroscopy to directly identify adsorption geometries, the solvent-switch outcome provides an experimental constraint indicating that the dominant DMSO effect is not permanent deactivation.

In IPA, FE values remained lower and more erratic (30–79%). CuNi achieved 79% FE with 58% GVL at −1.8 V, but in several cases, selectivity inverted toward VA (e.g., Cu at −1.6 V, 53% FE with 58% VA). The relatively high viscosity and low permittivity of IPA likely hinder ion dissociation, thereby increasing the sensitivity of electroreduction outcomes to subtle variations in applied potential and interfacial state. Rather than promoting a consistent mechanistic pathway, IPA accentuates the vulnerability of the system to transport limitations and double-layer instabilities.

Across all solvents, a key trend emerges: conversion and FE are frequently decoupled. For example, Ni in MeOH at −1.6 V achieved 96% conversion but only 36% FE, with the majority of charge remaining unassigned (100 – summed FE (GVL+VA+4-HVA)). Conversely, Cu in IPA at −1.6 V reached 71% conversion and 53% FE, largely as VA. Such contrasts underscore that conversion alone exaggerates performance; FE reveals the true electron partitioning. Importantly, given our FE definition, the consistently large unassigned charge fraction (100 – summed FE (GVL+VA+4-HVA), 20–90%) serves as a warning flag for hidden sinks—most plausibly hydrogen evolution, solvent-activation pathways, and potentially volatile carbon species. While solvent/electrolyte coreduction or acid–solvent chemistry could in principle yield organic products, these species are not targeted here and may fall outside the scope of LC detection (e.g., volatile organics and gas-phase products). Accordingly, within the objective of LA-to-GVL production, these pathways are treated as yield losses and are conservatively reported as unassigned charge (and, on the molar side, as unquantified converted LA in the “Other products” selectivity term). Notably, the relative ranking of Cu, Ni, and CuNi is not conserved across solvents and applied potentials, and in several cases it even inverts. This variability indicates that solvent-defined factors—proton activity, *iR*/transport losses (viscosity/conductivity), and interfacial structuring (double-layer reorganization and competitive adsorption)—can dominate the observable performance hierarchy and obscure catalyst-intrinsic trends. Accordingly, mechanistic interpretations based solely on catalyst identity can be misleading in nonaqueous ECH, because the medium and operating point determine which pathways are kinetically accessible and how charge partitions between productive hydrogenation and parasitic sinks. Therefore, throughout this work we can interpret catalyst effects only within solvent-defined operating windows and anchor comparisons in controlled electrolysis metrics (conversion, FE, and product distribution) rather than voltammetric current magnitude.

At this stage, it is pertinent to ask: *what is catalyst-intrinsic and what is medium-imposed?* The present results suggest that solvent and temperature exert a stronger influence than catalyst identity in determining efficiency and selectivity. In principle, Cu, Ni, and CuNi should differ in hydrogen adsorption/activation and in C = O hydrogenation capacity. Indeed, there are cases where CuNi delivers the highest performance (e.g., MeOH at −1.8 V; IPA at −1.8 V; DMSO at −1.8 V). Yet the hierarchy is inconsistent: in MeOH at −1.6 V, glassy carbon surpasses CuNi (87% vs 69% FE; 35 °C). While CuNi and Ni frequently perform well, their apparent advantages vanish—or even invert—depending on the solvent environment. This indicates that mechanistic interpretations based solely on catalyst identity risk conflating solvent effects with intrinsic material properties. Solvent, potential, and temperature routinely mask—or reverse—catalyst trends by controlling proton availability, uncompensated resistance, double-layer structure, and thermally assisted product consolidation (HVA → GVL). In practice, what may appear as a “catalyst effect” can often be attributed to medium control.

A plausible mechanistic framework emerges from solvent-specific behavior:

- MeOH: High conductivity and low viscosity favor efficient charge delivery; high proton activity promotes surface H formation; at 35 °C, rapid HVA → GVL lactonization consolidates intermediates, increasing FE through product stabilization.
- DMSO: Despite its high dielectric constant, limited proton availability constrains hydrogenation; outer-sphere stabilization of polar intermediates can bias selectivity toward VA at certain potentials. Efficient outcomes occur only within narrow operational windows, where overpotentials are sufficiently moderated to avoid charge leakage.
- IPA: High viscosity, low permittivity, and ion pairing restrict carrier density and mass transport, rendering selectivity highly sensitive to potential and interfacial structure. Frequent shifts between VA- and GVL-dominated pathways, together with a persistent contribution of “Other products,” highlight the limitations of IPA as a medium.

In MeOH, FE on GC increases dramatically when shifting from −1.8 to −1.6 V (53 → 87%), whereas Ni shows the opposite trend, collapsing from 79 to 36%. In DMSO, Cu remains inefficient at both potentials, while CuNi exhibits a pronounced improvement at −1.6 V, achieving near-complete product closure. As a result, the optimal operating point emerges as both medium- and material-dependent, rather than following a universal potential–efficiency relationship.

In summary, the electrocatalytic hydrogenation of LA in nonaqueous solvents is governed as much by the medium as by the catalyst. From the selected solvents, methanol provides the most favorable balance of conductivity, viscosity, and proton availability, enabling high FE and GVL selectivity once lactonization becomes thermally accessible. DMSO supports selective pathways under moderated potentials but suffers from charge leakage outside narrow operating windows, while IPA imposes persistent transport and ion-dissociation penalties that destabilize selectivity. Catalyst effects are detectable, particularly for CuNi, yet their expression is strongly conditioned by solvent and potential, to the point that apparent hierarchies can be inverted. These results highlight that medium engineering is also a decisive variable in nonaqueous ECH, but also a confounding one: improvements in FE may reflect product consolidation or suppressed HER as much as intrinsic electron-transfer kinetics. A robust mechanistic picture will require explicit quantification of all charge sinks, including gas-phase and volatile products, together with interfacial diagnostics capable of disentangling solvent-induced transport from catalyst-intrinsic reactivity.

#### Mechanistic Hypothesis for LA Hydrogenation and Temperature-Gated Lactonization

To rationalize the product distributions and the experimentally observed temperature switch from HVA to GVL, we outline a working mechanistic framework for the ECH of LA under nonaqueous conditions (Figures 4 and 5).^10,11^ The pathway proceeds via two conceptually simple, sequential steps: (i) electrochemical reduction of LA to HVA, and (ii) intramolecular lactonization of HVA to GVL. At high cathodic bias, (iii) overhydrogenation of HVA or ring-opened intermediates can further generate VA:^48^

- Step (i): LA → HVA: In the first step, hydrogenation of the carbonyl group occurs, commonly rationalized through a Volmer–Tafel sequence: protons are reduced to chemisorbed hydrogen atoms (H_ads_) in the Volmer step, which either hydrogenate LA to HVA or recombine to form H_2_ (Tafel pathway). From this perspective, low FE at 15 °C reflect a competition for H_ads_ between productive hydrogenation and the HER. The production of HVA at low temperature aligns with this view: the electrochemical step is accessible, but the subsequent chemical step (ring closure) is slow. Yet, this interpretation is not without weaknesses. On Cu electrodes, where H binding is intrinsically weak, invoking a Volmer–Tafel sequence is problematic; proton–electron transfer may proceed instead through concerted proton–electron transfer (CPET) or solvent-assisted mechanisms, particularly in DMSO and IPA where H_ads_ stability is poor. In fact, the modest FE and solvent dependence across Cu systems caution against assuming a universal H_ads_-driven mechanism. While the Volmer–Tafel framework provides a reasonable description for Ni and CuNi in MeOH, its applicability across all media and electrode materials appears restricted.
- Step (ii): HVA → GVL: Once HVA is formed, its ring closure to GVL is thermodynamically favorable (ΔG° ≈ −13.6 kJ mol^–1^, DFT at 298 K), but strongly kinetically limited under mild conditions. Consistent with this, product distributions show predominant 4-HVA accumulation at 15 °C with negligible GVL, whereas at 35 °C GVL becomes the dominant product. These trends indicate that 4-HVA is not a thermodynamic sink but a kinetic intermediate, and that GVL selectivity is governed by the thermal accessibility of the lactonization barrier. Importantly, an increased FE to GVL at higher temperature should be interpreted as enhanced downstream consolidation of intermediates into the counted target product; by itself, it does not prove reduced parasitic charge consumption (e.g., HER), which must be assessed independently via overall FE closure and/or gas quantification. Structurally, only γ-lactonization is relevant in this pathway: formation of a six-membered δ-valerolactone would require a 5-hydroxy acid precursor that is not generated along the LA → 4-HVA hydrogenation sequence. Therefore, the practical selectivity challenge is balancing kinetic access to lactonization (4-HVA → GVL) against 4-HVA accumulation at low temperature and potential over-reduction pathways at excessive cathodic bias.
- Step (iii): Over-reduction and VA formation: Valeric acid appears sporadically, particularly under strongly cathodic conditions or in solvents that destabilize lactonization. Two routes are plausible: (1) direct overhydrogenation of HVA’s hydroxyl group, or (2) reduction of ring-opened GVL intermediates. Both require higher H_ads_ coverage or alternative proton–electron coupling channels, explaining why VA is more pronounced at high bias and in solvents where GVL is unstable. This pathway is critical because it represents a charge sink that consumes electrons without generating the target cyclic product.

**Figure 4:**
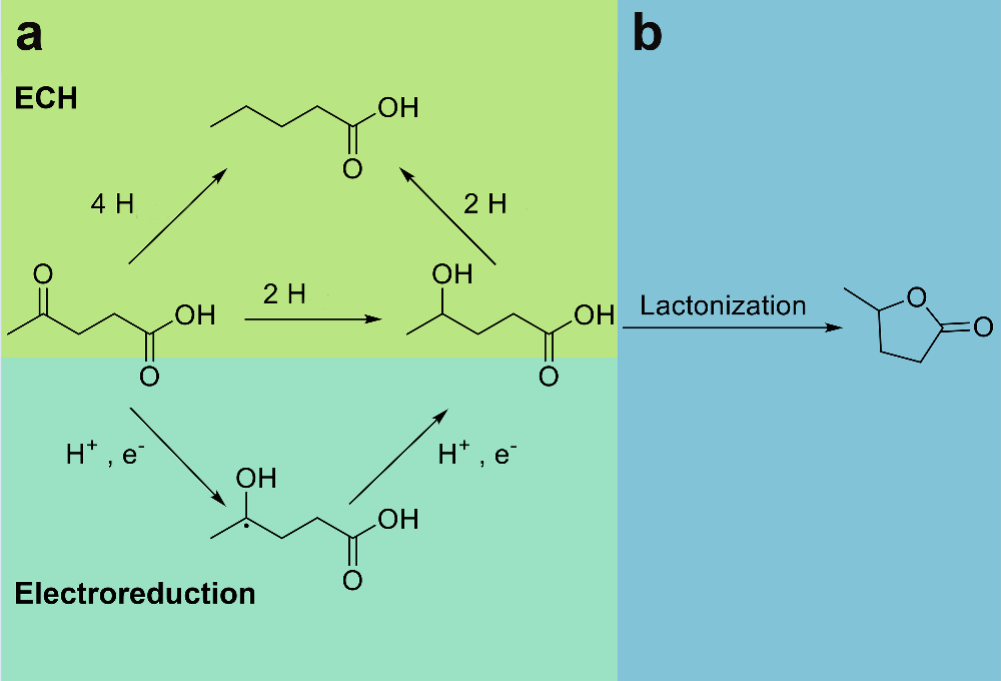
Proposed mechanistic pathway for the electro–thermal conversion of LA to GVL. (a) ECH of LA to HVA via H transfer. (b) Thermally assisted intramolecular lactonization of HVA in solution, yielding GVL + H_2_O.

**Figure 5:**
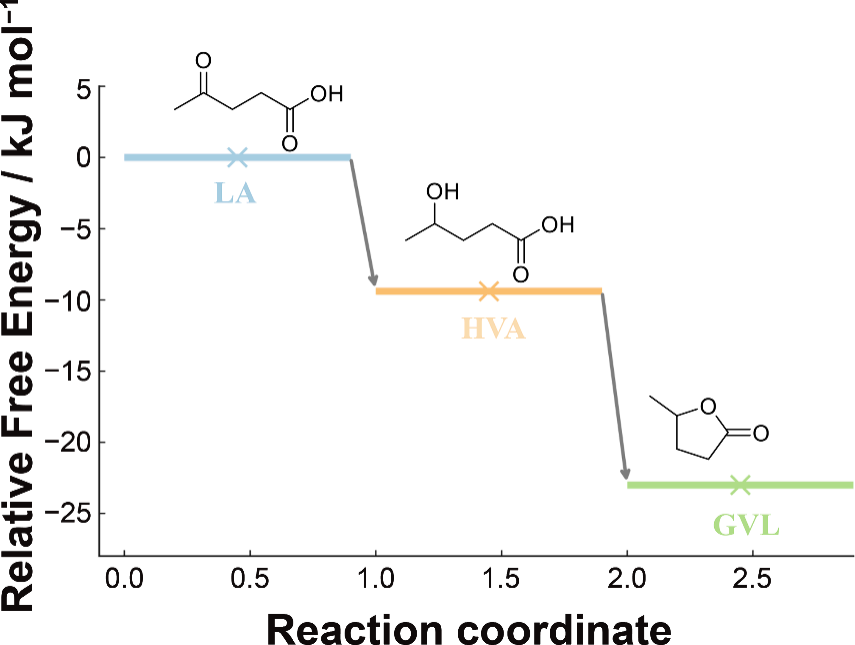
Reaction free-energy profile for LA → HVA → GVL. Schematic thermodynamic free-energy diagram showing the two elementary steps: (i) electrochemical hydrogenation of LA to HVA with ΔG° = −9.4 kJ mol^–1^ and (ii) thermally/acid-assisted lactonization of HVA to GVL+ H_2_O with ΔG° = −13.6 kJ mol^–1^ (overall ΔG° ≈ −23 kJ mol^–1^ at 298 K). ΔG° values refer to relative Gibbs free energies of stationary states; activation barriers are not included.

In order to rationalize the experimentally observed product distributions and the temperature-dependent switch from HVA to GVL, standard Gibbs free energies (ΔG°) were computed for the key molecular species involved (LA, HVA, GVL, H_2_O, H_2_) using DFT with frequency analysis at 298 K (Figure 5 and Table S7). All reaction free energies were obtained from isolated species thermochemistry at the ωB97X-D/6-31G* level, including vibrational (ZPE/thermal) corrections, with solvation treated using SMD implicit solvent model. Reactant and product free energies were combined stoichiometrically (ΔG° = ∑G°products – ∑G°reactants). Importantly, HOMO–LUMO gaps are not used in this work to infer kinetics or reaction barriers; rather, the DFT analysis is used to quantify ground-state thermodynamic driving forces for the proposed steps. Accordingly, the “reaction profile” in Figure 5 reports relative Gibbs free energies of stationary states (reactants/intermediates/products) and does not provide activation barriers. While explicit solvent-phase transition-state calculations (and, ideally, an electrochemical interface/constant-potential framework) would be required for quantitative barrier prediction, the experimentally observed temperature dependence is most consistently interpreted as kinetic gating: hydrogenation is comparatively facile, whereas lactonization requires thermal assistance, in line with Arrhenius/Eyring expectations, and is not dictated by thermodynamic preference alone.

The first reduction step, LA → HVA, is predicted to be mildly exergonic (ΔG° = −9.4 kJ mol^–1^), confirming that this transformation is thermodynamically feasible under electrochemical hydrogenation conditions. The experimentally observed HVA accumulation at 15 °C is consistent with a step that is not thermodynamically limited but rather governed by kinetic and transport constraints. At lower temperatures, limited ionic mobility, higher solution viscosity, and reduced exchange current densities slow down electron–proton transfer to the carbonyl, leading to incomplete utilization of the applied charge. Raising the temperature improves ionic transport (Stokes–Einstein), lowers viscosity, and accelerates charge-transfer rates, thereby enhancing the production of HVA. Thus, this step benefits kinetically from elevated T even though its thermodynamic driving force is modest but sufficient.

The second step, HVA → GVL + H_2_O, is more exergonic (ΔG° = −13.6 kJ mol^–1^), yielding an overall ΔG° ≈ −23 kJ mol^–1^ for LA → GVL at 298 K. Although thermodynamics favor this transformation, the experimental data reveal that lactonization is strongly temperature dependent. At 15 °C, HVA emerges as the dominant product, suggesting that the cyclization step is limited by a significant kinetic barrier. In contrast, at 35 °C the lactonization reaction is activated, leading to a sharp increase in GVL selectivity and FE. Within an Arrhenius/Eyring framework, this behavior indicates that HVA is a kinetic intermediate, stabilized at low temperature but readily converted to GVL once the activation barrier for ring closure can be overcome. Acidic conditions introduced by H_2_SO_4_ may further assist lactonization, but the need for thermal input remains evident.

The mechanistic framework outlined here—electrochemical reduction of LA to HVA followed by thermally assisted lactonization to GVL—provides an appealingly simple narrative. However, the experimental evidence points to a more intricate reality in which competing hydrogenation routes, solvent-driven transport effects, and unquantified electron sinks strongly influence the product slate. The broader message is pragmatic: solvent properties exert greater control over efficiency and selectivity than catalyst identity, yet this leverage comes with interpretive pitfalls and questions of scalability. Medium engineering can open selective operating windows, but the intrinsic activity of the catalyst cannot be fully resolved until complete electron balances, mechanistic diagnostics, and process-relevant assessments are incorporated.

#### Scale-Up and Process Outlook

While the present study targets fundamental mechanistic disentanglement (solvent versus temperature effects) rather than process optimization, translating LA-to-GVL electrocatalysis toward industrial relevance will require addressing coupled barriers in energy efficiency, electrolyzer design, catalyst durability, and separations. A primary constraint in nonaqueous operation is the reduced ionic conductivity compared with aqueous electrolytes, which increases *iR* losses and can limit attainable current densities at acceptable cell voltages. In this context, our measured solvent-dependent conductivity/viscosity trends and the markedly longer electrolysis times in viscous media motivate the use of thin-gap or zero-gap flow architectures (e.g., MEA-like or narrow interelectrode-gap flow cells) and 3D porous electrodes to increase areal productivity while minimizing ohmic and mass-transport penalties.^49,50^

Long-term durability under continuous operation is another key requirement. Relevant failure modes include to prevent metal dissolution/corrosion in strongly acidic electrolytes, catalyst restructuring under sustained bias, and performance drift due to interfacial fouling by solvent/electrolyte-derived adsorbates. Although the solvent-switch recovery control indicates no persistent deactivation after DMSO exposure under our conditions, validating process stability will require extended time-on-stream testing under flow and postoperation diagnostics (e.g., composition/leaching and surface/morphology checks).

Finally, separation and integration will be decisive in overall cost. Maximizing GVL selectivity while suppressing VA/HVA and unquantified sinks simplifies downstream purification and solvent/electrolyte recycle. Process-intensification strategies may include integrated solvent recovery and supporting-electrolyte management (e.g., membrane/electrodialysis or extraction-based recycle loops), and pairing the cathodic hydrogenation with value-added anodic reactions to replace oxygen evolution reaction (OER), thereby lowering full-cell voltage while generating a saleable anodic product stream. Such “value-added electrolysis” concepts are increasingly recognized as a key lever for improving techno-economic feasibility and accelerating integration into biomass-derived chemical value chains.

## Conclusions

This study establishes that in the electrocatalytic hydrogenation of levulinic acid, the solvent is not a neutral carrier but a primary design variable that governs efficiency, selectivity, and mechanistic accessibility. By systematically comparing methanol, isopropanol, and dimethyl sulfoxide across glassy carbon, Cu, Ni, and CuNi electrodes, we show that solvent properties—viscosity, dielectric constant, ionic conductivity, and proton availability—influence the operational boundaries far more strongly than the nominal catalyst identity.

Methanol provided the most permissive environment, combining high ionic conductivity and low viscosity with sufficient proton activity to sustain hydrogenation. Once the thermally activated HVA → GVL lactonization step became accessible at 35 °C, Faradaic efficiencies reached 80% with GVL as the dominant product. DMSO offered isolated conditions of high efficiency and unusually clean product distributions, but only under narrowly tuned potentials; elsewhere, its high viscosity and weak proton availability amplified charge leakage into side reactions. Isopropanol consistently penalized charge efficiency, with selectivity flipping between VA and GVL depending on potential and surface state, underscoring its transport and ion-dissociation limitations.

Across all solvents, conversion and Faradaic efficiencies were frequently decoupled. High conversions at low FE revealed that substantial fractions of the applied charge were consumed by hidden sinks, most plausibly hydrogen evolution, solvent-activation pathways, and volatile carbon species. This highlights the risk of overstating process performance by reporting conversion alone. Moreover, the intuitive expectation that less negative potentials should suppress HER and enhance FE was not consistently validated: potential, temperature, and solvent interacted nonlinearly, producing trends that were medium- and surface-specific rather than universal.

Mechanistically, the pathway LA → HVA → GVL is thermodynamically downhill, but the HVA → GVL lactonization step is kinetically gated. At 15 °C, production of HVA reflects the inability to overcome this barrier, whereas at 35 °C lactonization proceeds, consolidating electrons into GVL and inflating FE. Sporadic valeric acid formation further indicates that over-reduction and ring-opening reactions compete at high cathodic bias, representing additional charge sinks. Importantly, invoking a Volmer–Tafel hydrogenation mechanism is plausible for Ni and CuNi in methanol, but less convincing for Cu or for polar aprotic solvents, where weak H adsorption may necessitate alternative pathways such as CPET or solvent-assisted hydrogenation.

The broader implications are both promising and cautionary. Solvent engineering can suppress HER, stabilize reactive intermediates, and direct product selectivity, opening operational windows that deliver impressive FE and high GVL selectivity with non-noble catalysts. Yet these gains may reflect product consolidation or solvent control rather than intrinsic catalytic improvements, raising questions of scalability and robustness. The persistent dominance of “other products” underscores the need for full electron balances—including gas and volatile fractions—as well as in situ interfacial probes capable of disentangling transport phenomena from genuine catalyst activity.

In practical terms, three design rules emerge:

- Solvent sets the ceiling—methanol provides the broadest window, DMSO yields selective regimes only under narrow conditions, and IPA imposes severe transport penalties.
- Temperature is a gate—lactonization is thermally activated, so apparent efficiency gains at 35 °C reflect kinetic accessibility rather than improved electron utilization.
- Potential is a lever, not a guarantee—its effect on selectivity is medium- and surface-dependent, often nonlinear, and cannot be generalized.

Ultimately, catalyst-intrinsic activity is only revealed within narrow, solvent-defined windows. Unlocking its true potential will require integrated strategies: quantifying all charge sinks, probing interfacial fields and solvation structures, and designing process-relevant electrolytes and reactors. Only with these tools can medium control be transformed from a confounding factor into a deliberate strategy for scalable biomass (electro)­valorization. From a process standpoint, MeOH also offers a practical advantage as a volatile medium that can be readily recovered/recycled, so maximizing GVL selectivity (while minimizing VA and unquantified sinks) directly simplifies downstream enrichment and purification of the GVL-containing product fraction.

Finally, scaling this technology toward industrial relevance will require addressing coupled techno-economic barriers in energy efficiency, reactor architecture, and separations. Because organic electrolytes generally exhibit lower ionic conductivity than aqueous media, mitigating ohmic losses will be essential; this motivates a transition from batch cells to thin-gap/zero-gap flow electrolyzers (e.g., MEA-like architectures) to minimize *iR* penalties while enabling higher areal productivities. In parallel, downstream processing must prioritize solvent and supporting-electrolyte recycle—for example via membrane-enabled recovery (electrodialysis/ion-exchange) and/or extraction-based loops—to reduce operating cost and simplify purification. From a process perspective, the solvent/temperature design rules established here translate into clear scale-up targets: selecting high-conductivity, low-viscosity media to alleviate transport and *iR* limitations, and operating in windows that maximize GVL selectivity to minimize purification burden. These considerations also motivate coupling the cathodic LA-to-GVL conversion with value-added anodic reactions (in place of OER) as a route to lower full-cell energy demand and improve overall process economics.

## Supplementary Material



## Notes

Experimental and computational details; compositions of the electrodeposition baths and organic electrolytes; electrolysis parameters, average durations, and cathodic current responses for all electrode–solvent pairs; postelectrolysis XPS and EDS characterization; Faradaic efficiency calculation procedure and discussion of unassigned charge fractions and minor byproduct pathways; time-resolved NMR spectra of 4-HVA incubation experiments; and DFT-derived thermochemical parameters for all key molecular species (PDF)
