# Supplementary material for: Decoding Solvent Effects in Electrocatalytic Biomass Valorization: Levulinic Acid to γ‑Valerolactone
Source: ACS Sustain Chem Eng. 2026 Apr 8;14(20):9407–22. doi: 10.1021/acssuschemeng.5c12833 (PMC13220161; doi:10.1021/acssuschemeng.5c12833)
Supplement: Supplementary file 1 [file sc5c12833_si_001.pdf]

Supporting Information

Decoding Solvent Effects in Electrocatalytic Biomass Valorization: Levulinic Acid to Gamma-Valerolactone

Pol Vilariño[1,2], Queralt Bautista[1,2], Elvira Gómez [1,2] and Albert Serrà [1,2,*]

[1] Grup d'Electrodeposició de Capes Primes i Nanoestructures (GE-CPN), Departament de Ciència de Materials i Química Física, Universitat de Barcelona, Martí i Franquès, 1, E-08028, Barcelona, Catalonia, Spain.

[2] Institute of Nanoscience and Nanotechnology (IN^{2}UB), Universitat de Barcelona, Barcelona, Catalonia, Spain.

Corresponding author: a.serra@ub.edu (A.S.)

Bath compositions used for electrodeposition of Ni, Cu, and CuNi catalysts

Table S1. Electrodeposition bath compositions for Ni, Cu, and CuNi catalysts

	**Ni**	**Cu**	**CuNi**
**$CuCl_2$ / M**	0.00	0.05	0.05
**$NiCl_2$ / M**	0.30	0.00	0.30
**$C_6H_5Na_3O_7$ / M**	0.20	0.20	0.20
**NaCl / M**	0.20	0.95	0.05

Electrolyte Preparation and Composition

Table S2. Electrolyte compositions used in electrochemical experiments. Solutions were prepared in DMSO, IPA, or MeOH with and without levulinic acid (LA). Blank samples contain all components except LA.

	**DMSO - LA**	**DMSO - Blank**	**IPA - LA**	**IPA - Blank**	**MeOH - LA**	**MeOH - Blank**
**Solvent**	Dimethyl Sulfoxide	Dimethyl Sulfoxide	2-propanol	2-propanol	Methanol	Methanol
**LA / M**	0.50	-	0.50	-	0.50	-
**H_2SO_4 / M**	0.50	0.50	0.50	0.50	0.50	0.50
**Et_4NBF_4 / M**	0.10	0.10	0.10	0.10	0.10	0.10

Cathodic Current Response in LA Versus Blank Electrolytes

Table S3. Difference in cathodic current density between LA-containing and blank electrolytes ($\Delta j = j_{LA} - j_{Blank}$, μA cm^{-2}) for Cu, Ni, GC, and CuNi electrodes in MeOH, IPA, and DMSO at 15 °C and 35 °C.

**Δj (LA–Blank) / μA cm^{-2}**	**Cu 15 °C**	**Ni 15 °C**	**GC 15 °C**	**CuNi 15 °C**	**Cu 35 °C**	**Ni 35 °C**	**GC 35 °C**	**CuNi 35 °C**
**IPA (-1.6 V)**	-9.0	-0.2	-0.2	-1.5	-3.3	-7.7	-1.5	2.5
**DMSO (-1.6 V)**	-4.7	-1.5	-2.2	-6.0	-0.7	1.3	-6.0	-0.2
**MeOH (-1.6 V)**	-19.5	-14.2	-19.3	-24.9	-7.9	-10.0	-24.9	-38.8
**IPA (-1.8 V)**	-10.7	-3.8	-0.9	-2.0	-4.5	-9.8	-2.0	-1.5
**DMSO (-1.8 V)**	-12.6	-1.5	-4.3	-5.0	-1.6	-2.4	-5.0	-4.8
**MeOH (-1.8 V)**	-22.5	-18.0	-27.2	-29.1	-22.8	-25.8	-29.1	-45.7

Electrolysis Conditions in Non-Aqueous LA Solutions

Table S4. Electrolysis conditions for 10 mL LA solutions in MeOH, IPA, and DMSO. Each electrolysis was performed with a total passed charge of 1000 C.

**Solvent**	**Total Passed Charge / C**	**Volume / mL**	**Average electrolysis duration / h**
**MeOH**	1000	10	<10
**IPA**	1000	10	~20
**DMSO**	1000	10	~40

Post-electrolysis Stability and Surface Chemical State of CuNi Electrode

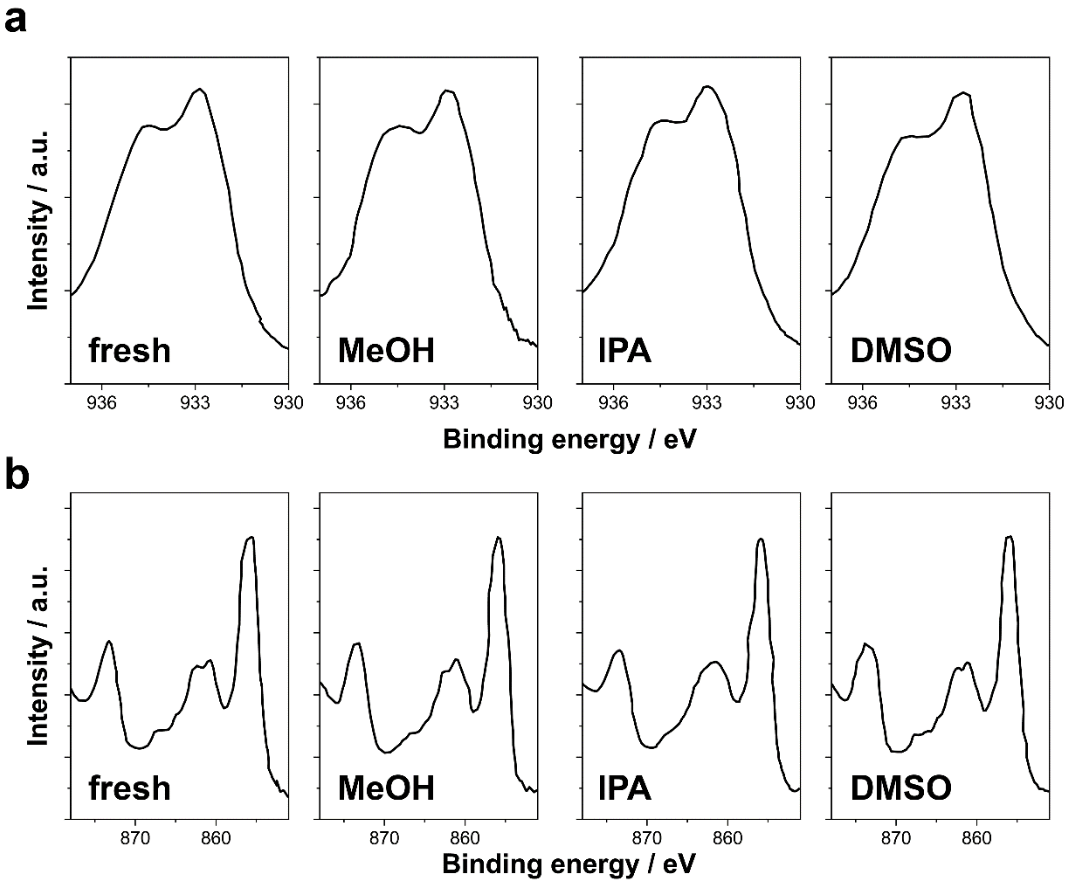

Figure S1. XPS spectra of the CuNi electrodes in the (a) Cu 2p and (b) Ni 2p regions before (pristine) and after electrolysis in MeOH, IPA, and DMSO under the standard fixed-charge protocol. No discernible changes in binding energy positions or line shapes are observed after electrolysis, indicating preservation of the near-surface chemical state across solvents within the explored operating window.

Faradaic Efficiency (FE)

The Faradaic efficiency (FE) for the electrochemical hydrogenation (ECH) of levulinic acid (LA) was calculated using:

$$\boldsymbol{FE}(\%) = \sum\left(\frac{Mole\ of\ produced\ product\ i \times n_i \times F}{Total\ charge\ passed}\right) \times 100$$

where n_i is the number of electrons required to form each quantified product (2 for γ-valerolactone (GVL) and 4-hydroxyvaleric acid (HVA); 4 for valeric acid (VA)), FFF is the Faraday constant (96485 C mol^{-1}), and Q_{total} is the total charge passed during electrolysis (in coulombs). Minor byproducts were not included in this calculation.

All electrolysis were performed under a fixed passed charge of Q = 1000 C (**Table S4**), corresponding to Q/F = 10.36 mmol e^-. With 5.0 mmol of LA, this protocol therefore supplies 2.07 e^- per LA molecule. The first hydrogenation step (LA → 4-HVA, and thus the net transformation LA → GVL, given that 4-HVA → GVL is a non-faradaic lactonization) is a 2 e^- process; complete faradaic conversion of 5.0 mmol LA through this step would require approximately 965 C. Consequently, if LA depletion is dominated by the 2 e^- hydrogenation step, high conversions necessarily leave limited charge headroom for the hydrogen evolution reaction (HER). As an upper bound, the maximum charge available for HER after achieving a conversion X (expressed as a fraction of LA_0) is:

$$Q_{HER,\,max} = Q - 2F(X - LA_0)$$

$$n(H_2)_{max} = \frac{Q_{HER,\,max}}{2F}$$

For example, at 90% conversion, $n(H_2)_{max} \leq$ 0.68 mmol; at 50% conversion, $n(H_2)_{max} \leq$ 2.68 mmol. Therefore, the unassigned charge fraction [100−∑FE(GVL+VA+4 HVA)] cannot be attributed uniquely to HER; it can also arise from unquantified organic electroreduction products and/or concurrent non-faradaic LA consumption (e.g., acid-catalyzed esterification in alcohol media, ketalization, and/or adsorption/retention phenomena).

Importantly, the product distribution columns reported in **Tables S5–S6** (GVL, VA, 4-HVA, and “Other products”) are molar selectivities (i.e., % of converted LA). Accordingly, “Other products” represents the fraction of converted LA not recovered as the three quantified products; it does not denote a charge fraction.

Beyond the primary target (GVL) and the quantified intermediate/products (4-HVA and VA), electrocatalytic hydrogenation of a multifunctional substrate such as LA in acidic non-aqueous media can generate a complex network of parallel and consecutive reactions (**Figure S2**). A fraction of LA may be diverted toward minor derivatives (e.g., levulinate/valerate esters in alcohol solvents, ethers and cyclic ethers such as 2-methyltetrahydrofuran, polyols such as 1,4-pentanediol, and fragmented/over-reduced chains), which may not be fully resolved by the single LC method used here. Exhaustive identification and quantification of these species would require a dedicated analytical campaign combining $^{1}H/^{13}C$ NMR, GC–MS, and orthogonal LC detection (e.g., multidimensional LC and/or UV/RI detection) to deconvolute overlapping signals from compounds with similar polarity and molecular weight. Because the present study is strategically focused on establishing medium-engineering rules for GVL production, complete speciation of all minor byproducts is considered outside the scope. Accordingly, these species are conservatively treated as yield losses and are included within the "Other products" selectivity term and/or within the unassigned charge fraction.

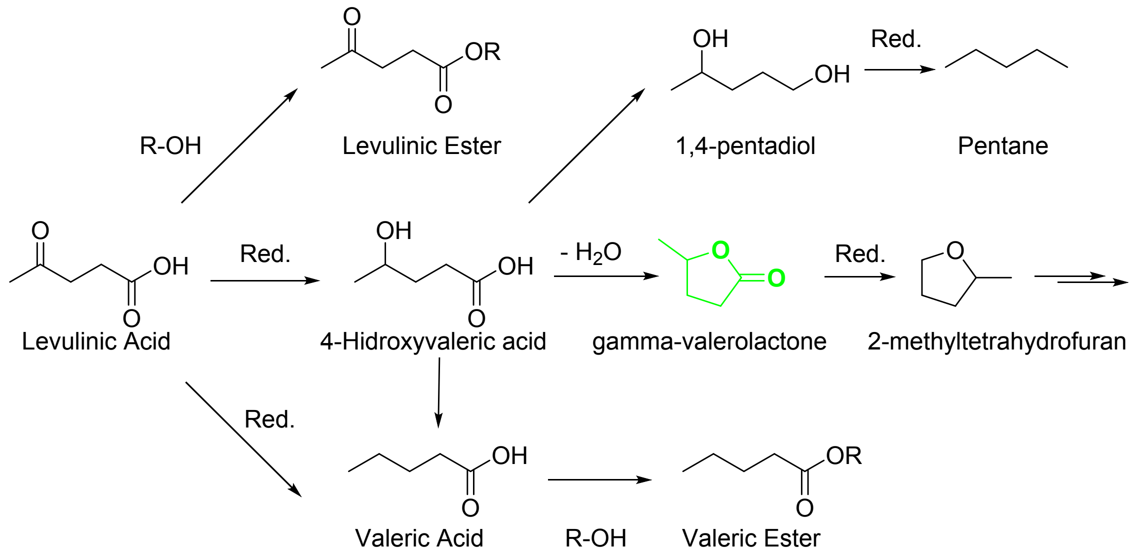

Figure S2. Plausible parallel and sequential reaction pathways for LA hydrogenation in acidic non-aqueous media, highlighting representative minor byproducts (e.g., levulinate/valerate esters, 2-methyltetrahydrofuran, 1,4-pentanediol, pentane, and related over-reduction/fragmentation products).

Electrocatalytic Reduction of LA

Table S5. Conversion percentages and product distribution (GVL, VA & HVA) obtained for different catalysts (GC, Cu, Ni, CuNi) in various solvents (IPA, DMSO, MeOH) at applied potentials of –1.6 V and –1.8 V. All experiments were performed at 15 °C.

Catalyst	Solvent	Applied Potential / V	Total charge / C	LA_0 / mmol	Conversion / %	FE / %	GVL / %	VA / %	HVA / %	Other products / %
**GC**	IPA	-1.8	1000	5.0	50.0 ± 2.1	3.1 ± 0.2	0.0 ± 0.0	0.0 ± 0.0	6.4 ± 0.3	93.6 ± 0.3
**GC**	IPA	-1.6	1000	5.0	53.0 ± 3.3	4.3 ± 0.1	0.0 ± 0.0	0.0 ± 0.0	8.5 ± 0.3	91.5 ± 0.6
**GC**	DMSO	-1.8	1000	5.0	64.0 ± 1.7	5.5 ± 0.2	0.0 ± 0.0	0.0 ± 0.0	8.9 ± 0.4	91.1 ± 0.2
**GC**	DMSO	-1.6	1000	5.0	60.0 ± 2.1	5.2 ± 0.3	0.0 ± 0.0	0.0 ± 0.0	9.0 ± 0.3	91.0 ± 0.3
**GC**	MeOH	-1.8	1000	5.0	83.1± 2.2	8.0 ± 0.2	0.0 ± 0.0	0.0 ± 0.0	10.0 ± 0.4	90.0 ± 0.1
**GC**	MeOH	-1.6	1000	5.0	80.4 ± 2.0	7.6 ± 0.1	0.0 ± 0.0	0.0 ± 0.0	9.9 ± 0.3	90.1 ± 0.3
**Cu**	IPA	-1.8	1000	5.0	36.3 ± 1.7	4.1 ± 0.3	0.0 ± 0.0	0.0 ± 0.0	10.8 ± 0.2	89.2 ± 0.2
**Cu**	IPA	-1.6	1000	5.0	39.9 ± 1.8	3.7 ± 0.2	0.0 ± 0.0	0.0 ± 0.0	10.6 ± 0.3	89.4 ± 0.1
**Cu**	DMSO	-1.8	1000	5.0	59.0 ± 1.1	5.8 ± 0.2	0.0 ± 0.0	0.0 ± 0.0	10.2 ± 0.3	89.8 ± 0.3
**Cu**	DMSO	-1.6	1000	5.0	53.6 ± 1.9	5.5 ± 0.1	0.0 ± 0.0	0.0 ± 0.0	10.8 ± 0.3	89.2 ± 0.2
**Cu**	MeOH	-1.8	1000	5.0	85.5 ± 1.5	8.8 ± 0.2	0.0 ± 0.0	0.0 ± 0.0	10.7 ± 0.4	89.3 ± 0.1
**Cu**	MeOH	-1.6	1000	5.0	86.4 ± 1.4	8.8 ± 0.1	0.0 ± 0.0	0.0 ± 0.0	10.2 ± 0.5	89.8 ± 0.3

**Ni**	IPA	-1.8	1000	5.0	36.2 ± 1.6	3.8 ± 0.2	0.0 ± 0.0	0.0 ± 0.0	10.8 ± 0.2	89.2 ± 0.2
**Ni**	IPA	-1.6	1000	5.0	33.3 ± 1.0	4.2 ± 0.2	0.0 ± 0.0	0.0 ± 0.0	13.3 ± 0.3	86.7 ± 0.2
**Ni**	DMSO	-1.8	1000	5.0	57.8 ± 1.5	5.3 ± 0.1	0.0 ± 0.0	0.0 ± 0.0	9.5 ± 0.3	90.5 ± 0.1
**Ni**	DMSO	-1.6	1000	5.0	55.5 ± 1.6	5.7 ± 0.3	0.0 ± 0.0	0.0 ± 0.0	10.6 ± 0.3	89.4 ± 0.3
**Ni**	MeOH	-1.8	1000	5.0	88.3 ± 2.6	8.6 ± 0.2	0.0 ± 0.0	0.0 ± 0.0	10.1 ± 0.4	89.9 ± 0.2
**Ni**	MeOH	-1.6	1000	5.0	91.3 ± 3.6	9.0 ± 0.1	0.0 ± 0.0	0.0 ± 0.0	10.2 ± 0.5	89.8 ± 0.1
**CuNi**	IPA	-1.8	1000	5.0	59.1 ± 1.8	5.9 ± 0.3	0.0 ± 0.0	0.0 ± 0.0	10.3 ± 0.3	89.7 ± 0.3
**CuNi**	IPA	-1.6	1000	5.0	61.2 ± 2.2	6.2 ± 0.2	0.0 ± 0.0	0.0 ± 0.0	10.5 ± 0.3	89.5 ± 0.2
**CuNi**	DMSO	-1.8	1000	5.0	51.6 ± 2.0	5.0 ± 0.1	0.0 ± 0.0	0.0 ± 0.0	10.1 ± 0.3	89.9 ± 0.3
**CuNi**	DMSO	-1.6	1000	5.0	50.5 ± 1.1	4.8 ± 0.1	0.0 ± 0.0	0.0 ± 0.0	8.2 ± 0.3	91.8 ± 0.1
**CuNi**	MeOH	-1.8	1000	5.0	91.2 ± 1.5	8.7 ± 0.2	0.0 ± 0.0	0.0 ± 0.0	9.9 ± 0.4	90.1 ± 0.2
**CuNi**	MeOH	-1.6	1000	5.0	90.9 ± 1.4	8.4 ± 0.2	0.0 ± 0.0	0.0 ± 0.0	9.5 ± 0.4	90.5 ± 0.1

Table S6. Conversion percentages and product distribution (GVL, VA & HVA) obtained for different catalysts (GC, Cu, Ni, CuNi) in various solvents (IPA, DMSO, MeOH) at applied potentials of –1.6 V and –1.8 V. All experiments were performed at 35 °C.

Catalyst	Solvent	Applied Potential / V	Total charge / C	LA_0 / mmol	Conversion / %	FE / %	GVL / %	VA / %	HVA / %	Other products / %
**GC**	IPA	-1.8	1000	5.0	87.2 ± 1.9	35.2 ± 0.3	15.1 ± 2.3	13.4 ± 1.2	0.0 ± 0.0	71.5 ± 0.9
**GC**	IPA	-1.6	1000	5.0	87.5 ± 1.7	47.6 ± 1.3	38.6 ± 1.3	8.9 ± 1.2	0.0 ± 0.0	52.5 ± 1.1
**GC**	DMSO	-1.8	1000	5.0	77.6 ± 2.6	52.8 ± 0.8	28.9 ± 2.1	20.9 ± 1.8	0.0 ± 0.0	50.3 ± 0.6
**GC**	DMSO	-1.6	1000	5.0	85.1 ± 1.9	58.1 ± 1.1	46.1 ± 2.3	47.7 ± 1.1	0.0 ± 0.0	6.2 ± 0.9
**GC**	MeOH	-1.8	1000	5.0	87.5 ± 2.1	53.0 ± 0.4	24.8 ± 1.9	50.3 ± 2.0	0.0 ± 0.0	24.8 ± 0.6
**GC**	MeOH	-1.6	1000	5.0	89.9 ± 2.3	87.4 ± 0.4	74.8 ± 2.9	13.0 ± 2.5	0.0 ± 0.0	12.2 ± 1.1
**Cu**	IPA	-1.8	1000	5.0	70.9 ± 2.0	41.0 ± 2.1	47.0 ± 2.9	6.5 ± 1.2	0.0 ± 0.0	46.5 ± 2.2
**Cu**	IPA	-1.6	1000	5.0	70.9 ± 2.7	52.9 ± 1.1	38.1 ± 2.3	58.3± 2.6	0.0 ± 0.0	3.6 ± 1.3
**Cu**	DMSO	-1.8	1000	5.0	86.8 ± 2.2	17.1 ± 0.2	10.6 ± 1.4	4.9 ± 2.1	0.0 ± 0.0	84.5 ± 0.3
**Cu**	DMSO	-1.6	1000	5.0	89.5 ± 2.6	19.0 ± 0.6	11.7 ± 2.8	5.1 ± 1.7	0.0 ± 0.0	83.2 ± 0.9
**Cu**	MeOH	-1.8	1000	5.0	84.2 ± 2.1	76.7 ± 1.1	83.7± 2.6	5.5 ± 1.8	0.0 ± 0.0	10.9 ± 1.1
**Cu**	MeOH	-1.6	1000	5.0	71.3 ± 2.0	69.5 ± 0.1	70.7 ± 1.6	15.1 ± 1.1	0.0 ± 0.0	14.1 ± 0.3
**Ni**	IPA	-1.8	1000	5.0	87.5 ± 2.6	62.5 ± 0.7	38.7 ± 2.1	17.6 ± 2.2	0.0 ± 0.0	43.6 ± 0.8
**Ni**	IPA	-1.6	1000	5.0	75.8 ± 1.8	56.1 ± 1.2	5.3 ± 2.7	35.7 ± 2.4	0.0 ± 0.0	59.0 ± 1.2

**Ni**	DMSO	-1.8	1000	5.0	82.1 ± 2.4	46.8 ± 1.1	48.1 ± 2.2	5.6 ± 2.0	0.0 ± 0.0	46.4 ± 2.1
**Ni**	DMSO	-1.6	1000	5.0	87.2 ± 2.9	70.7 ± 0.7	28.8 ± 1.9	27.6 ± 2.7	0.0 ± 0.0	43.6 ± 2.3
**Ni**	MeOH	-1.8	1000	5.0	95.2 ± 2.3	79.1 ± 0.4	73.7 ± 2.5	6.3 ± 1.9	0.0 ± 0.0	20.0 ± 0.7
**Ni**	MeOH	-1.6	1000	5.0	96.1 ± 2.7	35.9 ± 0.1	18.4 ± 2.0	10.2 ± 1.5	0.0 ± 0.0	71.4 ± 1.1
**CuNi**	IPA	-1.8	1000	5.0	85.1 ± 2.8	78.9 ± 0.9	58.1 ± 2.5	19.0 ± 1.7	0.0 ± 0.0	22.9 ± 1.3
**CuNi**	IPA	-1.6	1000	5.0	71.3± 2.6	30.5 ± 0.8	31.5± 2.0	6.4 ± 1.6	0.0 ± 0.0	62.1 ± 1.2
**CuNi**	DMSO	-1.8	1000	5.0	77.6 ± 2.3	71.8 ± 1.2	26.1 ± 2.2	34.9 ± 2.7	0.0 ± 0.0	39.0 ± 0.7
**CuNi**	DMSO	-1.6	1000	5.0	86.8 ± 2.8	60.9 ± 1.4	50.2 ± 1.5	47.6 ± 2.0	0.0 ± 0.0	2.2 ± 0.9
**CuNi**	MeOH	-1.8	1000	5.0	87.5 ± 2.5	79.8 ± 1.2	76.8 ± 2.7	8.9 ± 1.9	0.0 ± 0.0	14.3 ± 2.1
**CuNi**	MeOH	-1.6	1000	5.0	89.5 ± 2.1	69.2 ± 1.6	69.9 ± 1.9	5.1± 2.4	0.0 ± 0.0	25.0 ± 1.1

Temperature- and solvent-dependent lactonization of 4-hydroxyvaleric acid (4-HVA)

To experimentally test whether the temperature dependence of GVL selectivity originates from the homogeneous lactonization of the 4-HVA intermediate (rather than catalyst deactivation or electrochemical transport effects), we performed electrode-free probe experiments using analytical-grade 4-HVA under the same acid/solvent/supporting-electrolyte composition employed for electrolysis. This "molecular decoupling" approach isolates the cyclodehydration step (4-HVA → GVL) from the electrochemical interface, enabling an unambiguous assessment of how temperature and solvent affect lactonization.

Mechanistically, the cyclization of 4-HVA into GVL is an intramolecular nucleophilic acyl substitution (**Figure S3**). The hydroxyl oxygen at the C4 position must undergo a conformational reorientation to perform a nucleophilic attack on the C1 carboxylic carbon. In the presence of a strong acid catalyst (H_2SO_4), the carboxylic group is protonated to enhance its electrophilicity. However, the formation of the intermediate and the subsequent elimination of water require the system to overcome an entropic and enthalpic threshold. At a reduced temperature of 15 °C, the open-chain 4-HVA intermediate undergoes significant stabilization through intermolecular hydrogen-bonding networks in protic media (MeOH/IPA). These interactions geometrically constrain the molecule in its linear conformation, effectively elevating the energy barrier for cyclization. Similarly, in aprotic polar solvents such as DMSO, strong dipole-dipole interactions further rigidify the open-chain structure, hindering the conformational reorientation necessary for lactonization.

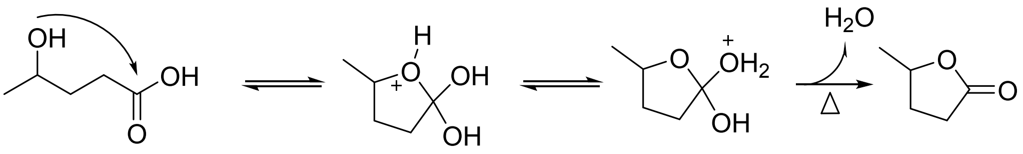

Figure S3. Proposed reaction mechanism and kinetic pathways for the electrocatalytic hydrogenation of LA to GVL.

The ^1H NMR spectra (**Figure S4**) recorded at the 8-hour mark for each temperature provide a comprehensive narrative of the "gate-opening" process. At 15 °C, the spectrum remains completely static, mirroring the baseline of the pristine 4-HVA probe. The dominant signals are a multiplet at δ 3.85 ppm (the methine proton, –CH–OH) and a doublet at δ 1.15 ppm (the terminal methyl group). The total absence of lactone signals in this spectrum proves that at 15 °C, the kinetic barrier is, effectively halting the reaction at the intermediate stage. This is a critical

discovery, as it physically explains why GVL yields are negligible at this temperature even if the catalyst successfully reduces the LA substrate.

As the temperature is incrementally raised to 20 °C, 25 °C, and 30 °C, the 8-hour spectra reveal the progressive resolution of the kinetic blockage. A new resonance begins to emerge at δ 4.52 ppm, representing the methine proton as it shifts downfield due to the deshielding effect of the newly formed ester oxygen in the lactone ring. The intensity of this peak grows systematically with each 5-degree increase, illustrating the gradual "sampling" of the transition state. By 30 °C, the conversion is notable but not yet dominant, indicating that while the gate is "unlocked," the rate of cyclization remains relatively slow compared to the 35 °C target.

Finally, at 35 °C, the 8-hour spectrum displays a dramatic shift in the chemical equilibrium toward GVL. The signal at δ 4.52 ppm becomes the primary peak for the methine proton, while the methylene ($–CH_2–$) protons shift significantly to the 2.0–2.6 ppm region, reflecting the conformational rigidity of the cyclic structure. Additionally, the methyl doublet shifts downfield to δ 1.41 ppm. The integration of these resonances confirms that at 35 °C, the thermal energy is optimal for overcoming the cyclization barrier, leading to the high GVL selectivities reported in our FE tables (**Table S4** and **S5**).

Finally, a critical spectroscopic marker of the reaction's progress is the resonance observed at δ 11.0–12.0 ppm, characteristic of the carboxylic acid proton (–COOH). In the 8-hour spectrum at 15 °C, this signal remains prominent, confirming the structural integrity of the open-chain 4-hydroxyvaleric acid intermediate. However, as the temperature is increased to 35 °C, this peak undergoes a complete disappearance. This loss of the acidic proton is the direct result of the intramolecular dehydration process, providing definitive evidence of the successful cyclization into the lactone ring.

This exhaustive NMR study serves as the experimental anchor for our entire mechanistic proposal. It proves that the "missing" yield at lower temperatures is not due to catalyst failure or solvent degradation, but to a physical kinetic trap where the intermediate (4-HVA) simply cannot cyclize. By aligning these spectroscopic findings with our DFT models and the macroscopic HPLC data, we can confidently state that there is a thermo-electrochemical hybrid process. The catalyst's role is to provide the electrons for the initial reduction, but the Medium Engineering (specifically the management of temperature and solvent-solute interactions) is part of what ultimately dictates the successful production of GVL. This insight allows us to move beyond simple catalyst screening and toward the rational design of complete reaction environments for sustainable chemistry.

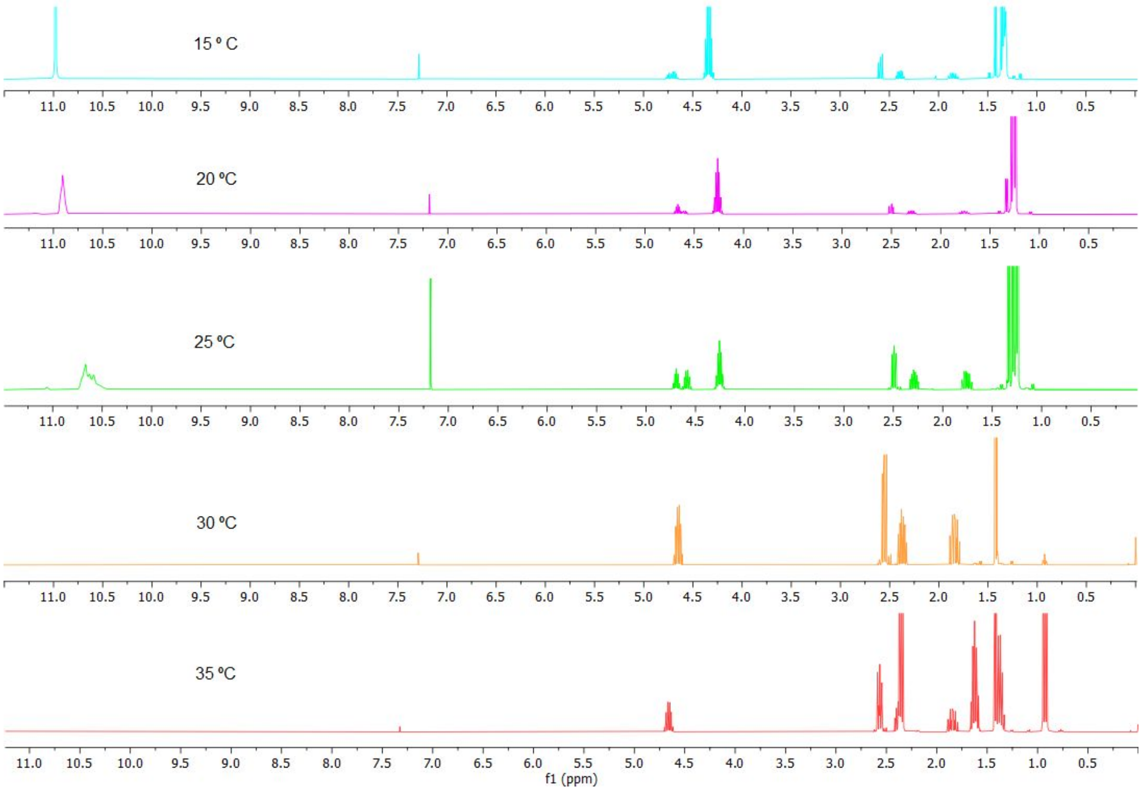

Figure S4. Experimental validation of the thermal kinetic gate via ^{1}H NMR spectroscopy. Time-resolved ^{1}H NMR spectra (400 MHz) of 4-hydroxyvaleric acid (4-HVA) incubation in the electrolytic medium (IPA + 0.5 M H_2SO_4) at various temperatures. At 15 °C, the spectra remain static over 8 h, showing exclusively the open-chain 4-HVA signals (δ 3.85 and 1.15 ppm). Spectra recorded at 20 °C, 25 °C, and 30 °C illustrate the progressive thermal activation of the system, with the gradual emergence of the GVL methine resonance. At 35 °C, a dominant conversion to GVL is observed within 8 h, marked by the prominent downfield shift of the methine signal to δ 4.52 ppm and the methylene protons to 2.0–2.6 ppm.

Thermochemical Parameters from DFT Calculations

Table S7. Thermochemical properties of key molecules involved in the electrocatalytic hydrogenation of levulinic acid (LA) obtained from DFT calculations at 298.15 K. Reported values include molecular formula, number of conformers (Confs), point group symmetry, dipole moment (μ, D), zero-point energy (ZPE, kJ·mol^{-1}), enthalpy (H°, Eh), entropy (S°, J·mol^{-1}·K^{-1}), Gibbs free energy (G°, Eh), and heat capacity (C_v, J·mol^{-1}·K^{-1}).

**Molecule**	**Formula**	**Confs**	**Point group**	**μ / D**	**ZPE / kJ·mol^{-1}**	**H° / Eh**	**S° / J·mol^{-1}·K^{-1}**	**G° / Eh**	**C_v / J·mol^{-1}·K^{-1}**
**GVL**	$C_5H_8O_2$	1	C1	4.63	318.10	−345.580520	329.38	−345.617924	104.95
**LA**	$C_5H_8O_3$	27	C1	2.44	322.56	−420.778571	361.32	−420.819603	132.18
**HVA**	$C_5H_{10}O_3$	81	C1	2.23	382.34	−421.954784	372.08	−421.997037	142.69
**VA**	$C_5H_{10}O_2$	27	Cs	1.48	371.48	−346.770250	352.07	−346.810231	124.29
**H_2O**	H_2O	1	C_2v	2.15	53.61	−76.362873	188.70	−76.384301	25.15
**H_2**	H_2	1	$D_{\infty}h$	0.00	25.14	−1.159051	130.23	−1.173839	20.79
